# Rigid multipodal platforms for metal surfaces

**DOI:** 10.3762/bjnano.7.34

**Published:** 2016-03-08

**Authors:** Michal Valášek, Marcin Lindner, Marcel Mayor

**Affiliations:** 1 Institute of Nanotechnology, Karlsruhe Institute of Technology (KIT), Hermann-von-Helmholtz-Platz 1, D-76344 Eggenstein-Leopoldshafen, Germany; 2 Lehn Institute of Functional Materials (LIFM), Sun Yat-Sen University (SYSU), Xingang Rd. W., Guangzhou, China; 3 Department of Chemistry, University of Basel, St. Johannsring 19, CH-4056 Basel, Switzerland

**Keywords:** multivalent anchoring, protruding structure, spatial arrangement, tripodal platform

## Abstract

In this review the recent progress in molecular platforms that form rigid and well-defined contact to a metal surface are discussed. Most of the presented examples have at least three anchoring units in order to control the spatial arrangement of the protruding molecular subunit. Another interesting feature is the lateral orientation of these foot structures which, depending on the particular application, is equally important as the spatial arrangement of the molecules. The numerous approaches towards assembling and organizing functional molecules into specific architectures on metal substrates are reviewed here. Particular attention is paid to variations of both, the core structures and the anchoring groups. Furthermore, the analytical methods enabling the investigation of individual molecules as well as monomolecular layers of ordered platform structures are summarized. The presented multipodal platforms bearing several anchoring groups form considerably more stable molecule–metal contacts than corresponding monopodal analogues and exhibit an enlarged separation of the functional molecules due to the increased footprint, as well as restrict tilting of the functional termini with respect to the metal surface. These platforms are thus ideally suited to tune important properties of the molecule–metal interface. On a single-molecule level, several of these platforms enable the control over the arrangement of the protruding rod-type molecular structures (e.g., molecular wires, switches, rotors, sensors) with respect to the surface of the substrate.

## Introduction

Molecular electronics, as motivated in the 1970s from a rather theoretical point of view by Kuhn and Möbius [1] and later by Aviram and Ratner [2], tries to get molecules wired and explore their potential use as electronic devices, logic gates or sensing entities [3–5]. The understanding of the fundamentals of electron transport through the molecules is essential for the development and exploration of possible electronic components [6].

Since the first electrical measurements on benzene-1,4-dithiol molecules in 1997 [7] research in molecular electronics has progressed rapidly. The rapid growth of modern methods based on nanolithography and scanning-probe techniques enable one to study the electrical properties of single molecules [8–11]. Current methods and approaches to characterize the behavior of single molecules in metal–molecule–metal junctions [12] are so varied. The most common measurements on single molecular junctions are based on either electrochemical break junctions [13–15] or mechanically controlled break junctions (MCBJ) [7] as well as on scanning tunneling microscopy (STM) [9,16–17].

The ultimate goal of molecular electronics is to use assemblies of molecules or even single molecules as functional building blocks and to integrate them into electric circuits between the macroscopic electrodes, where a sufficiently strong binding between two terminal anchoring groups of the bridging molecule and the metal electrode is achieved. Only if this requirement is met, the control over the electronic properties of single-molecule devices becomes possible, which is of paramount importance for molecular devices. Furthermore, not only the effect of anchoring groups, but also accurate measurements of the molecular conductance over the functional core and molecular wire is crucial to fulfill requirements for molecular electronic devices.

At present, the field of molecular electronic is far from maturity. The influence of the junction geometry on the electrical characteristics of single molecules remains a fascinating and important area of research. Early experiments have shown that the electrical characteristics of junctions of symmetric molecules are not necessarily symmetric under bias voltage [18]. Furthermore, the same molecule can exhibit various conductance values [19], and its interface with the electrodes, which is usually determined by chemical anchoring groups, can have a large influence on its electrical properties [20]. Moreover, transistor-type devices from the same molecule have displayed fundamentally different transport characteristics [21–22].

The organization of the molecules within the junction is usually based on some sort of self-assembly using chemisorption or physisorption methods to form monomolecular layers between both electrodes. In many cases, either self-assembled monolayers (SAMs) [23] or Langmuir–Blodgett films (LB) [24–25] of organic molecules on a solid surface provide the order at the molecular level inside the junction to accomplish interface functionalization. The anchoring group, responsible for the direct contact between metal and the functional molecule, needs to be considered in terms of its mechanical stability and also regarding its electronic transparency (week or strong coupling). An ideal molecular anchoring group is expected to provide well-defined and reproducible binding, sufficiently strong anchoring between a molecule and metal surfaces, and should maintain a sufficient electron density of states close to the Fermi level to pass an electron or hole through the molecule (electronically transparent nature with relatively high conductance). Finally, a well-defined spatial arrangement of the tailor-made functional molecules on a solid surface is of paramount importance in the design of single-molecule devices.

So far, many anchoring groups such as thiols (–SH) [26–29], amines (–NH_2_) [15,26,30], phosphines [31], pyridines [9,32–35], selenols (–SeH) [36–39], fullerenes [40–42], isocyanides (–NC) [30,43–44], nitriles (–CN) [45–46], nitro (–NO_2_) [46], isothiocyanides (–NCS) [47], methyl sulfide (–SCH_3_) [31], dithiocarbamates (–NCS_2_) [48], carbodithiolates (–CS_2_H) [49–50], hydroxyl (–OH) [51], N-heterocyclic carbenes [52–53], and carboxylic acids (–COOH) [26,54] have been investigated and used to form electronic devices, and also the influence of anchoring groups on single-molecule conductance has been examined. Different anchoring groups possess different coupling strengths and contact geometries, which significantly affect the charge transport properties of the molecular junctions [55]. Nevertheless, these anchoring groups have been explored most frequently when attached to core structures that are not highly conjugated and exhibit poor conductivity (e.g., saturated alkanes) [26,30,56]. In contrast, highly conjugated systems [57–58] are more promising candidates for molecular electronic wires, which is evident from a few comparative studies of anchoring groups in conjugated systems [33,44,46,51]. In saturated structures, the resistance of the core molecule is higher and thus the anchoring effect is reduced. While organic π-conjugated systems are capable of more efficient charge transport along the molecular backbone due to the electron delocalization. This fundamental phenomenon is induced by the difference in the energy gap between the lowest unoccupied molecular orbital (LUMO) and highest occupied molecular orbital (HOMO). In conjugated systems this gap is smaller (about 3 eV) than the HOMO–LUMO gap of saturated molecules (about 7 eV) [17]. The conductance of a conjugated system depends on several factors, and not only the length of the conjugated system and its anchoring groups have a large influence on the conductance of the molecule, but also other factors such as, e.g., the topological connection (*ortho*, *meta* or *para*) or the torsion angle between subunits are important [59–62].

Moreover, flat delocalized π-systems have a tendency to spread with the entire π-surface over the substrate driven by van der Waals interactions. While delocalized π-systems are the ideal model compounds for numerous electronic and optical applications, a perpendicular arrangement with respect to the surface would be desired to profit from their properties. In optical experiments the quenching of molecular excited states is reduced by a perpendicular arrangement and in electronic applications a perpendicular arrangement is required to separate the π-system from the substrate and to profit from the entire dimension of the molecule. While for most optical set-ups the perpendicular arrangement is the only prerequisite, in electronic applications also the contact point of the molecule with the substrate, which defines the coupling between molecule and electrode (substrate), must be controlled.

**Figure 1:**
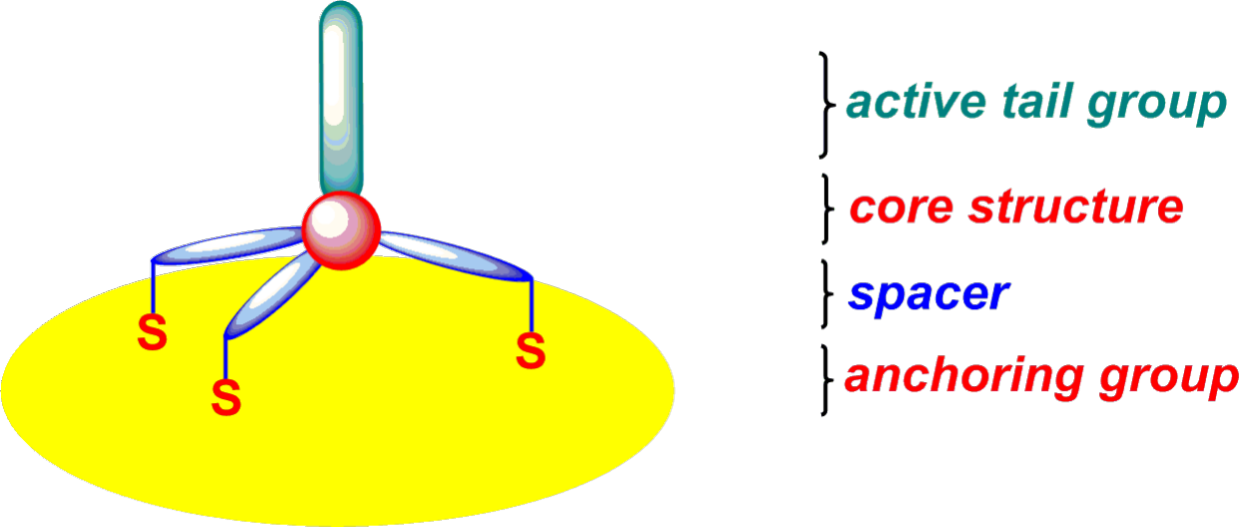
Schematic drawing of a molecule attached to the surface via a tripodal structure.

One of the most important class of SAMs is based on the strong chemisorption of organosulfur compounds (thiols, disulfides), and related moieties on coinage metals, particularly Au(111), Ag, Cu as well as Pt, Hg, GaAs(100) and InP(100) surfaces [23]. Particularly, the sulfur–gold bond is the most popular and the most extensively investigated junction for anchoring organic molecules on metal surfaces. Furthermore, there are several advantages of utilizing of gold as metal electrode for single molecule studies. One of the most important benefits of a gold substrate is that gold forms a reasonable clean, inert and atomically flat surface suitable for STM studies, which is not prone to impurities by reaction with oxygen and can be handled even under ambient conditions in the laboratory before its surface functionalization with organosulfur compounds. The covalent bond between sulfur and gold gives rise to robust and reasonably conductive single-molecular junctions of adsorbed molecules on gold substrates. Since the early stages of molecular electronics, most studies deal with molecules attached to the gold surface through one thiol (–SH) group [63]. While the details of the adsorption mechanism are still under debate, it is commonly considered that the hydrogen of the thiol group is eliminated in contact with gold and that a covalent Au–S bond is formed [23,64]. This bond has a dissociation energy of around 2.1 eV (ca. 50 kcal·mol^−1^), which is large enough to ensure the thermal stability of thiol monolayers up to 80 °C [65]. Furthermore, it is stronger than the Au–Au bond with a dissociation energy of around 0.8 eV [66], which can lead to the removal of small gold clusters by mechanically removing thiols. The versatility of the thiol anchoring guarantees a dense coverage of both flat and rough gold surfaces. The clean close packed Au(111) surface exhibits a hexagonal arrangement of atoms with a well-known long range 22 × √3 herringbone reconstruction with both face-centered cubic (fcc) and hexagonal close-packed (hcp) domains. But the absorption of sulfur-containing molecules (e.g., thiol, disulfide) on Au(111) forms a strong covalent bond and induces significant changes in the surface reconstruction of clean Au(111). This surface morphology changes related to adsorbed molecules can be visualized by STM techniques and provided us a reliable description of the interactions between adsorbate and substrate. Not only thiols but also sulfides (R–S–R), which form weaker molecule–substrate bonds than thiols, lift the herringbone reconstruction of Au(111) and remove a significant fraction of the gold atoms from the surface [67]. On the Au(111) surface thiols can bind to three sites, the so-called “top”, “bridge”, and “hollow” sites. In these configurations, the sulfur atom of the thiol is bound to one, two, or three gold atoms, respectively [68]. Furthermore, the high reactivity of the thiol group not only guarantees a robust functionalization of gold electrodes. It can also lead to complication during the self-assembly process. The intermolecular linking of bifunctional dithiols due to disulfide formation in the presence of trace amounts of oxygen may cause multilayer formation [69] and, in electrical measurements, the probing of disulfide oligomers. To address that problem the in situ formation of thiols from thioacetates with a deprotection agent can significantly reduce the risk of multilayer formation [63,70]. Although thiol monolayers have received considerable interest in the scientific community, the stability of these SAMs and the poor tolerability of Au in CMOS technology, considerably reduces the application potential. In particular, these organic films exhibit only moderate stability under ambient conditions and decompose at elevated temperatures. One of the drawbacks of thiol monolayers is that the molecular plane of absorbed thiols is inclined to the surface. Another drawback appeared in complex molecular systems that form densely packed SAMs, where the close spatial arrangement of neighboring molecules causes significant steric and/or electrostatic repulsions. To circumvent these problems, researchers have explored several strategies for generating thermally and chemically stable SAMs. Several approaches have been investigated to circumvent this problem, one of the most common protocol is employing mixed SAMs composed of two or more different molecules, where one has a longer alkyl chain than the others and carries a functional terminus. While this protocol is useful for molecules that form well-ordered and densely packed monolayers, it is ineligible to control the spatial arrangement and position of single molecules. New approaches to create free volume around the functional molecules in the monolayers and to achieve the effective electronic decoupling of individual molecules from the metal surfaces and to get high-performance molecular devices have been discovered recently. One of the most common protocols to increase the efficiency of single molecules bearing a sterically demanding functional tail in the self-assembled monolayers is based on employing either bulk spacer molecules or large multipodal platforms (Figure 1). Furthermore, the multipodal architecture also significantly increases binding stability of single molecules on metal surfaces.

In order to also control the spatial arrangement of the molecules rigid molecular architectures with multiple anchor groups are particularly appealing. Thus, the motivation for employing multipodal structures was to make a strong contact and to enforce an orientation of the molecules at a fixed distance from the surface [71]. A rigid multipodal architecture that guarantees a stable arrangement of single molecules on the surface is characterized by the presence of at least three anchoring groups that are not in a line. The basic platform that fulfills this criterion is a tripod, which is a common structure in chemistry. Chemical structures containing sp^3^-hybridized carbon or silicon core atoms represent a tripod with the fourth bond positioned perpendicular to the surface. The remaining three substituents of the tetrahedral core should be as rigid as possible to form a stable contact to the surface. Consequently, these legs usually contain rigid aromatic units or phenylene ethylene species if greater length is desired. So far, a number of *C*_3_-symmetric tripods incorporate a carbon atom (e.g., tetraphenylmethanes) [35,39,72–74], a silicon atom (e.g., tetraphenylsilanes) [75–80], or adamantane [81–87] as the branching unit decorated with three identical sulfur-containing termini (thiol, thioacetate, sulfides), selenol-containing termini or pyridine have been described and chemisorbed on gold surfaces. In these molecular tripods, which yield a stable and perpendicular chemisorption of molecules, however, little or no attention has been paid to maintaining the functionality of anchored molecules. In order to enable a fast electron transfer a strong and defined electronic coupling with the gold electrode is required. We note that tripodal adsorbates reported so far adopted anchors with aliphatic thiol groups that are not π-conjugated, such as benzylthiol and adamantylthiol. While some synthetic papers focused mainly on the concept [75–78,82], initial studies revealed an increased stability of the tripodal contact [72–73] and surface analysis by scanning probe methods [74,85–86] or X-ray absorption techniques [84,88–89] revealed an enlarged separation due to the increased footprint of the tripod. Further evidences for a perpendicular arrangement of separated molecules were obtained by optical [74,79,90] and electrochemical [80,87,89] analysis of the samples.

In addition, taking into account a well-defined alignment of the multipodal platforms on the surface, several groups have got more insight into its possible applications as a tip for scanning probe microscopy [75,81,91], a crosslinker for the creation of arrays of gold nanoparticles, and to anchor several active tail molecules as complex ligands [79–80], fullerenes [77–78,92], rotaxanes [93], pseudorotaxanes and artificial molecular rotors [94–97] to the surface. Although the most commonly used immobilization chemistry on gold electrodes is the formation of covalent bonds between thiols and gold substrates, also a few examples profit from the interaction of delocalized π-systems with the flat substrate to arrange a subunit perpendicular to the surface such as, e.g., the triazatriangulenium platforms from Herges and co-workers [98] or the tris(4-pyridyl-*p*-phenyl)methyl platform from Aso and co-workers [35].

While several of these multipods enable a perpendicular arrangement of rod-type molecular structures, the electronic coupling of the π-system of the rod to the metal states is limited due to the multipodal architectures comprising sp^3^-hybridized atoms. This electronic decoupling of the functional subunit is on one hand desired to profit from the optical properties of the subunit but on the other hand it represents a considerable handicap for molecular electronic applications.

In this review we discuss recent progress in multipodal platforms that form rigid and well-defined contact via at least three anchoring units to the metal surfaces (gold), and focus attention on the different core structures (aliphatic and aromatic systems) and anchoring groups. We also describe the emerging methods being used for the characterization of molecular junctions on the metal surfaces, and discuss the potential for the future research and applications. Finally, the authors apologize to their colleagues in the community for the strange wording describing their achievements. The rights of the copyright holders of the original research articles do not allow for a verbatim use of the original wording to describe the published results, a fact that considerably handicaps the precise reporting of the scientific achievements and thus also the writing of a review article.

## Review

### Aliphatic tripodal adsorbates

**Figure 2:**
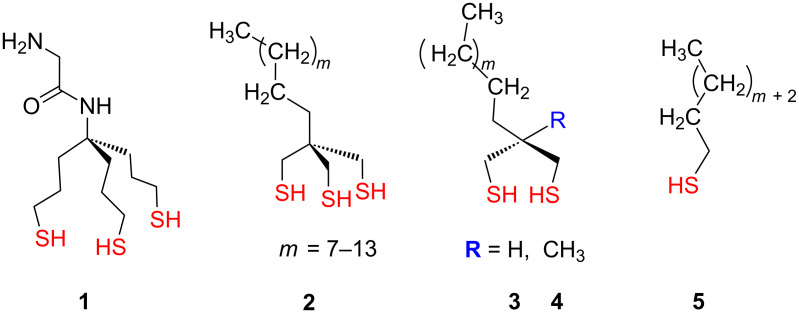
Aliphatic tripodal structures **1–5**.

Tripodal structures have been employed to engineer assemblies where three anchoring groups of a single platform can bind to the metal surface. First aliphatic aminotrithiol-based tripodal structure **1** (Figure 2) was introduced by Whitesell and Chang in 1993 [99]. They reported the controlled growth of α-helical peptides on a gold surface modified by this thiol-linking agent. Whitesell, Fox and co-workers used tris(3-sulfanylpropyl)methylamine derivative **1** as an effective linkage for binding surface probes (fluorescent or redox-active) that can be activated by light or by an applied potential on gold substrates [100]. Although **1** is an effective linking agent for binding surface probes, X-ray photoelectron spectroscopy (XPS) measurements revealed that monolayers of **1** are substantially disordered, with an average of 30% of the thiol groups not being bound to the gold surface, probably due to heavy steric interactions as suggested by molecular mechanics modelling.

Afterwards, Lee et al. introduced and synthetized a series of new tridentate chelating adsorbates **2** (Figure 2) having different alkyl chain lengths ranging from C12 to C18 and used them to prepare loosely packed self-assembled monolayers (SAMs) on gold [101]. The SAMs were characterized using ellipsometry, contact angle goniometry, polarization modulation-infrared reflection adsorption spectroscopy (PM-IRRAS) and XPS. The data in these fundamental studies were compared with those obtained from SAMs that formed by the adsorption of normal *n*-alkylthiols **5** and bidentate analogues **3**, **4** having similar chain lengths, to provide systematic control in packing density (Figure 2). The comparison showed that the SAMs of tridentate adsorbate **2** have lower packing densities of alkyl chains than the SAMs of bidentate **3**, **4** and monodentate **5** analogues. Consequently, the individual molecules in the SAMs of tridentate adsorbates **2** exhibit the least conformational order and the highest tilt from the surface. Additionally, an enhanced chelate effect of tridentate adsorbates **2** leads to a significant increase in the thermal stability of these SAMs than those derived from monodentate and bidentate adsorbates as revealed by preliminary studies at elevated temperatures using ellipsometry [102]. Later systematic studies of both thiol-functionalized flat gold surfaces and colloidal gold nanoparticles approved that the thermal stability of SAMs correlates with the degree of chelatation (i.e., tridentate > bidentate > monodentate) [103].

**Figure 3:**
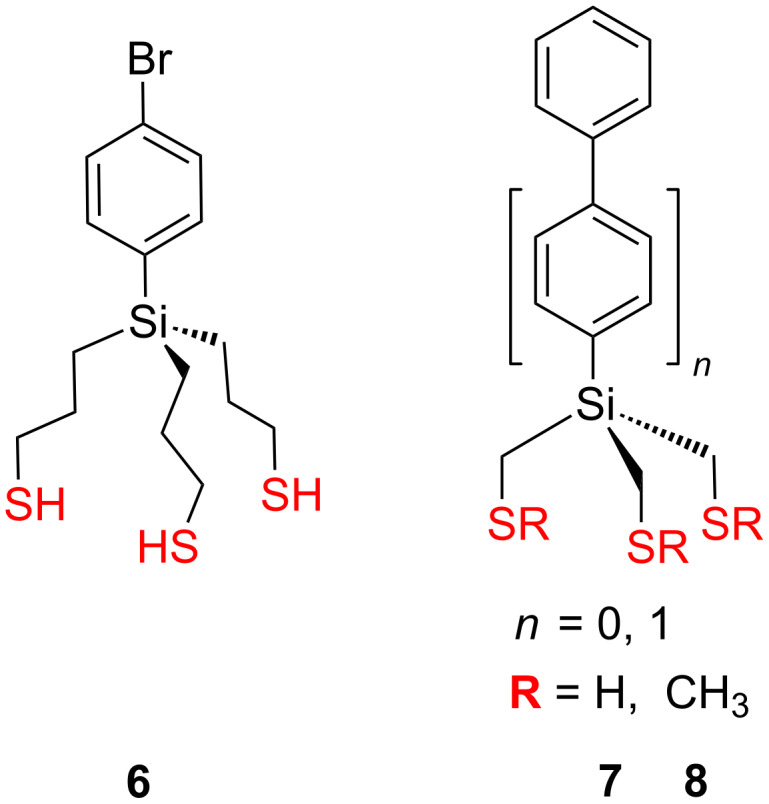
Trialkylarylsilane platforms **6–8**.

Trialkylarylsilane analogues have been also utilized for tripodal shape adsorbates. Cai and co-workers introduced the silicon trithiolate **6** [104], and formed SAMs on gold (Figure 3). As revealed by XPS measurements, approximately 20% of the terminal sulfur atoms were unbound to the gold surface, which is in agreement with the results obtained from the similar carbon-core aminotrithiol **1** [100]. Nevertheless, these films still possess greater stabilities in hot solvents compared with alkylthiol films due to the presence of multiple anchoring groups that enables strong binding of adsorbed molecules to the gold surface.

Recently, Weidner and co-workers have comprehensively studied a series of tridentate silane derivatives terminated with sulfanylmethyl **7** or methylsulfanylmethyl **8** groups (Figure 3) and used them for the fabrication of SAMs on gold [88,105]. In this study they particularly focused to reveal the surface properties of various sulfanylmethyl- and methylsulfanylmethyl-terminated tripodal platforms in order to get fairly densely packed, contamination free and homogeneous monolayers with a well-defined bonding configuration on gold substrates. Film formation from solution was investigated in situ by second harmonic generation (SHG) and ellipsometry, which revealed a two-step process (fast adsorption ≈ physisorption, followed by slow film ordering ≈ chemisorption). The SAMs were characterized by XPS, Fourier transform infrared absorption spectroscopy (FT-IRRAS), near-edge X-ray absorption fine structure spectroscopy (NEXAFS), and a scanning tunneling microscopy (STM) analysis. As revealed by XPS and NEXAFS analysis, the monolayers derived from the thiol-terminated adsorbates **7** exhibit significantly better packing density, molecular arrangement and binding uniformity than the corresponding methylsulfanyl-terminated analogues **8**. These results were supported by the XPS analysis, which revealed the presence of three different binding states of sulfur in the corresponding loosely packed films, commonly associated with weakly bound sulfur, unbound sulfur, disulfide moieties or a week coordination-type binding to the substrate. However, it should be noted that despite a better arrangement, a higher packing density and a significantly lower level of contaminations in the thiol-terminated adsorbate films of **7**, there is still a significant fraction of anchoring groups (approx. 35%) that are not bound to the gold surface.

**Figure 4:**
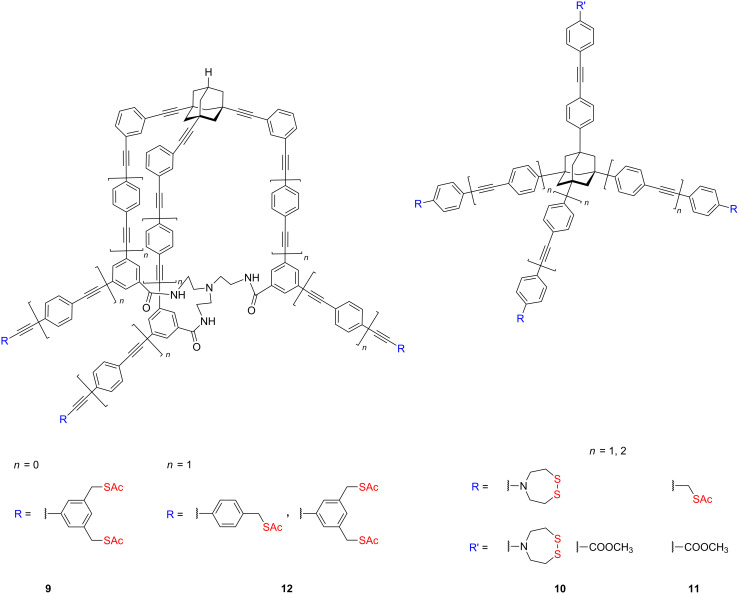
Structure of extended adamantane-based scaffolds **9–12**.

To improve a binding affinity of adsorbates to the metal substrates, several research groups have employed rigid platforms based on adamantane and cyclohexane moieties. The synthesis of adamantane-based tripodal platforms with sulfanylmethyl anchoring groups for chemisorption on gold was pioneered by Keana and co-workers [81–82,106]. The clearly defined geometry, the size and rigidity of sp^3^-hybridized tricyclic hydrocarbon scaffold as well as the easy functionalization at three of the bridgehead carbons, which allows for the attachment of the legs, have proven to be useful attributes for the surface application of 1,3,5,7-tetrasubstituted adamantane as one of the first rigid molecular platforms. Firstly Keana and co-workers synthetized a tower-shaped 3,5-bis(acetylsulfanylmethyl)phenyl-terminated adamantane moiety **9** (Figure 4) as an atomically sharp tip for atomic force microscopy (AFM) applications [91].

In the following studies they examined surface behavior of several 4-([1,2,5]-dithiazepan-5-yl)phenyl-terminated **10** and 4-(acetylsulfanylmethyl)phenyl-terminated **11** tetraphenyladamantanes (Figure 4) to get ideal AFM tip molecules, which should be composed of rigid molecules with well-defined geometry bearing terminal anchoring groups suitable for chemical functionalization of a commercial tip [81–106]. The anchoring platform of the ideal AFM tip molecule should be large enough and the total height of the tripodal scaffold should be such that neighboring molecules bound to the convex surface do not interfere with imaging by the apical molecules. When the commercial tip is covered with small molecular platforms, the final functionalization is leading only to increase of the radius of curvature of the molecular tip. Recently, two rigid, tower-shaped, tripodal nanoscale molecules **12** bearing three 4-(acetylsulfanylmethyl)phenyl and 3,5-bis(acetylsulfanylmethyl)phenyl feet designed for AFM applications have been synthetized and characterized (Figure 4). These novel molecules **12** are much larger versions of the prototypic molecule **9** and have a better aspect ratio, important for attachment to a commercial AFM tip. Furthermore, they showed that these macrocyclic trilactam moieties **12** terminated with 4-(acetylsulfanylmethyl)phenyl anchors are of sufficient size and rigidity to be visualized with a conventional AFM tip, as well as these well-defined bulk molecules may be further used for the calibration of AFM tips.

**Figure 5:**
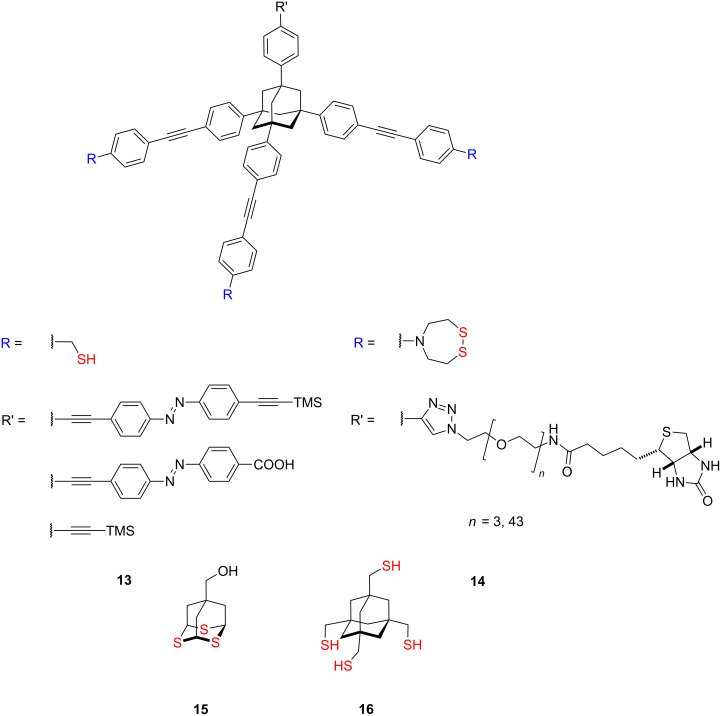
Structure of adamantane tripodal molecules **13–16**.

Extended adamantane-based tripodal molecules have been reported by Yamakoshi and co-workers [83,90]. They designed and examined azobenzene-terminated tripodal derivatives **13** (Figure 5), which are suitable as a single-molecular tip for noncontact atomic force microscopy (NC-AFM).

**Figure 6:**
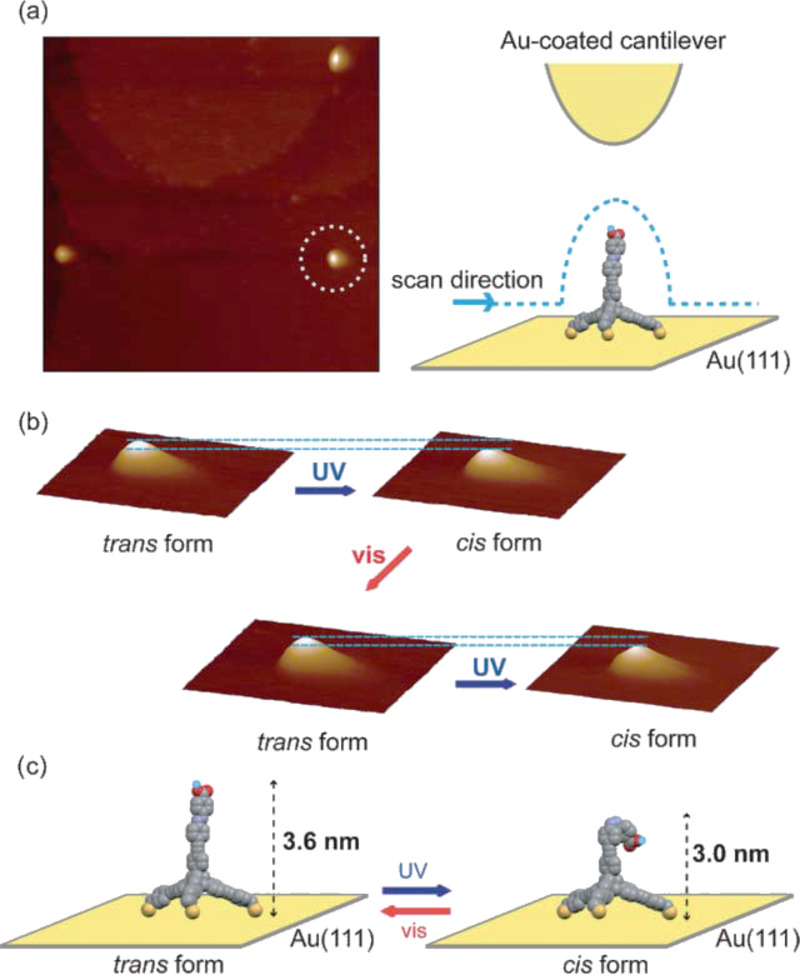
(a) UHV-NC-AFM image (350 × 350 nm^2^, Δ*f* = −28 Hz) of 4-carboxyazobenzene **13** adsorbed on Au(111) using a Au-coated cantilever and schematic of the measurement. (b) A series of 3D view of NC-AFM images (55 × 55 nm^2^, Δ*f* = −28 Hz) of **13** adsorbed on Au(111), which was indicated by a white circle in (a) representing the *trans* form (after visible light (450 ± 10 nm) irradiation) and the *cis* form (after UV light (360 ± 10 nm) irradiation) and corresponding line profiles are indicated. (c) Molecular models of **13** fixed on the Au substrate in *trans* and *cis* configurations. Reproduced with permission from [90], copyright 2010 The Royal Society of Chemistry.

The reversible photoisomerisation of these strongly bounded azobenzenes **13** with tripodal anchor mounted on the gold surfaces results in an in situ change of the tip apexes and in a radically different tip–sample interaction. These features not only allow for novel kinds of chemical analysis on submolecular scale but also enable high-resolution topographic imaging of the same sample surface by NC-AFM (Figure 6).

In an extended study they synthetized an acetylene-terminated adamantane tripod, which was easily functionalized with various ligand moieties by means of click chemistry at the terminal acetylene (Figure 5). They prepared two biotin-terminated tripodal tips **14**, which are useful for chemical force spectroscopy (CFS) measurements of the ligand–protein receptor interaction in a biotin–avidin model system, toward the development of high-throughput drug screening, and studies of transmembrane receptors [107]. Also, Whitesell and Fox during seeking more ordered surface layers synthesized the 2,4,9-trithiaadamantane derivative **15** and the 1,3,5,7-tetrakis(sulfanylmethyl)adamantane derivative **16** (Figure 5) and studied their binding properties on Au subsequently [84]. XPS studies show that, all three sulfur atoms of the sulfide moieties of **15** are bound to the gold surface, and that, on average, three of four thiols of **16** are chemisorbed onto gold surface.

**Figure 7:**
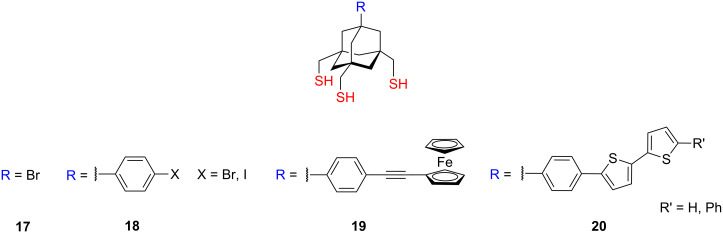
Adamantane-based tripods **17–20**.

But the major study dealing with the adamantane tripods was published by Kitagawa et al., who prepared and examined the chemisorption of the halogen-terminated adamantane tripods **17** and **18** (Figure 7) and firstly found that all three sulfur atoms of the bromine-terminated adamantane tripod **17** (1-bromo-3,5,7-tris(sulfanylmethyl)adamantane) were bound to the atomically flat Au(111) surfaces [85].

**Figure 8:**
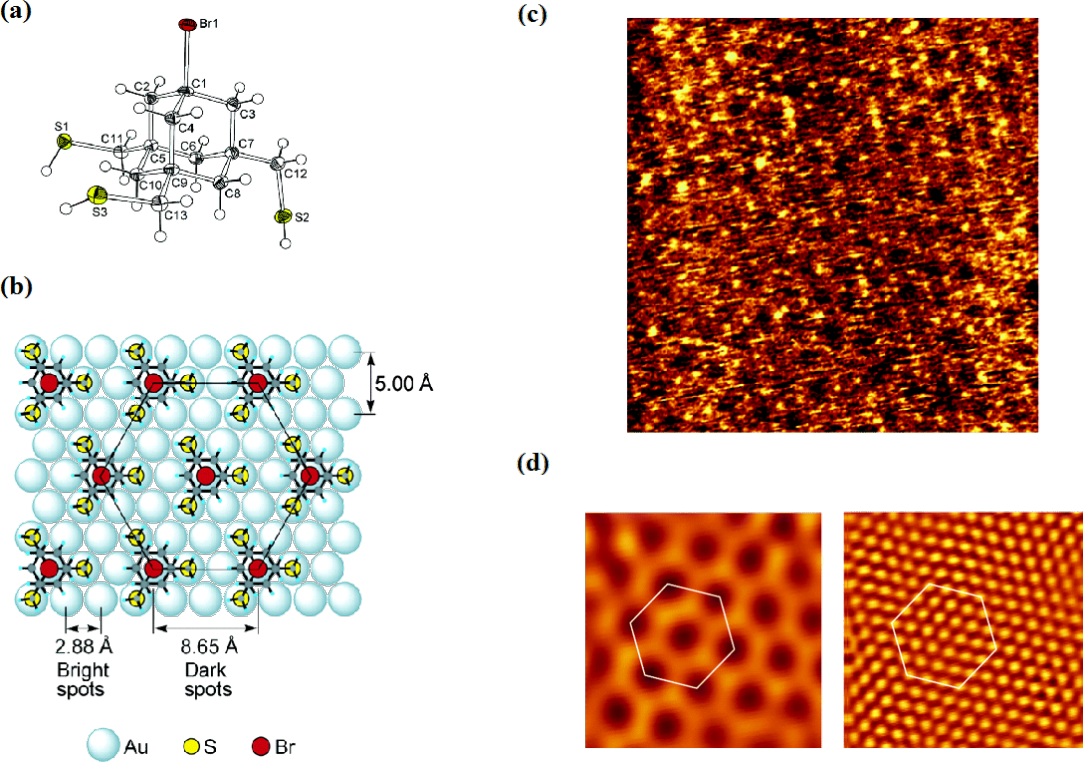
(a) ORTEP drawing of **17** as determined by single-crystal X-ray diffraction analysis at 100 K. Ellipsoids are drawn at the 50% probability level for non-hydrogen atoms. (b) Top view of a possible arrangement of **17** for the SAM on Au(111). The unit cell is shown by a hexagon. All molecules are drawn in the same orientation to minimize unfavorable intramolecular interactions. Sulfur atoms are assumed to be located on the near-top sites. (c) STM image of the SAM of **17** on a Au(111) surface, as measured in 0.1 M aqueous HClO_4_ at ambient temperature. A silver wire was used as the reference electrode. Image area 12 nm × 12 nm, set point current 400 pA, bias voltage 700 mV, Au electrode potential −300 mV, tip potential 400 mV. (d) Computer images of the 8.7 Å (left) and 2.9 Å (right) components of the lower left 4 nm × 4 nm area of (c). These images were obtained by inverse Fourier transform of each of the two intense frequency components obtained by two-dimensional Fourier transform of the raw image. The unit cell, indicated by hexagons, has a side length of 8.7 Å. Reprinted with permission from [85], copyright 2006 American Chemical Society.

This tripod is formed from three sulfanylmethyl feet that bond to the metal surface, by replacing the S–H bonds with S–metal bonds. The three-point chemisorption of these tripods was confirmed by PM-IRRAS, which showed the absence of a S–H stretching band at ca. 2570 cm^−1^. Furthermore, the initial STM analysis of SAMs prepared from **17** at the solid–liquid interface revealed the formation of two-dimensional crystal structures with a hexagonal arrangement of the adsorbed molecules on gold with a shortest intermolecular distance (lattice constant) of 8.7 Å (Figure 8). This distance allows for electroactive headgroups, which are linked to the SAM of adamantane tripods in the 1-position, to arrange in the same pattern on gold, if they are not too large to fit into the lattice. After anchoring to the gold substrate, these adamantane derivatives exhibit nearly anti-periplanar conformation of all three C–S bonds with respect to the C–Br bond with the expected intermolecular distance between neighboring sulfur atoms of about 5 Å, which closely matched the distance between sulfur atoms in the SAMs of alkanethiols on gold. This behavior ensures the concept of a rigid and well-defined arrangement of tripodal molecules with all three legs connected to the gold surface. The chemisorption of these tripods via almost all three sulfur legs was also confirmed by electrochemical reductive desorption experiments of the SAM of **17**, where the observed electric charge providing information on the surface concentration of the adsorbed molecules was in good agreement with the expected surface coverage, while the SAMs of 4-halophenyl derivatives **18** showed a somewhat smaller total reductive charge (ca. 70%). Also, the reduction peak potential of the SAM of **17** was shifted toward negative values compared with the SAMs of **18**, which was attributed to a strong bounding of the tripodal structure through three anchoring groups.

**Figure 9:**
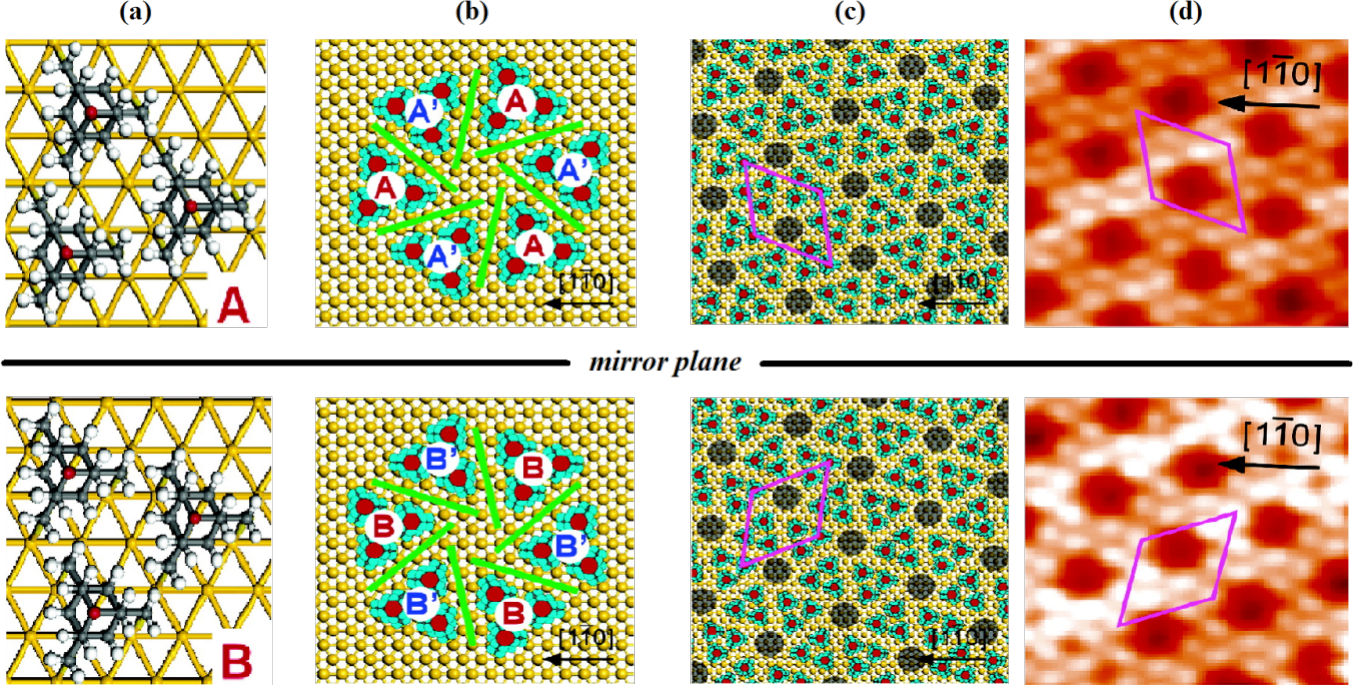
Schematic adsorption models of trimers (a), hexagon (b) and SAMs (c) of **17** on Au(111). B (B’) is the enantiomer of A (A’), the mirror plane of which is aligned parallel to the [1−10] direction. 180°-rotated configuration of A (B) is presented as A’ (B’). The chiral components of the trimers are indicated as A, A’, B, and B’. The hierarchical assembly is passed from trimer (a) to hexagon (b) and then to SAMs (c). Each enantiomeric hexagon is composed of the same chiral trimers as shown in (b). The unit cells of the SAMs are indicated in (c), whose periodicities are represented as 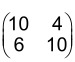 for A and 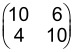 for B. (d) Corresponding STM images of monolayer SAMs (0.079 ML; 1 ML corresponds to the number of metal atoms on the bulk metal surfaces)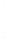 (*V*_s_ = −0.8 V, *I*_t_ = 0.3 nA). Reprinted with permission from [86], copyright 2007 American Chemical Society.

An extended study by Katano et al. investigated by UHV-STM analysis at 4.7 K also confirmed the three-point contacts of **17** on Au(111) surfaces and showed that these tripodal molecules form a highly ordered “two-tiered” hierarchical chiral self-assembly on a gold surface [86]. These achiral molecules are at first arranged on the surface in the form of chiral trimers, which then serve as the template for final 2D chiral hexagonal pattern. Upon adsorption on a gold surface, a racemic mixture of **17** self-assembles to form spatially ordered ribbon-like islands, which then lead to an enantiomeric domain and to hexagonal close-packed (hcp) sites. The enantiopure chiral subunits arrange into chiral trimers and further to hexagons to produce large-scale ordered chiral structures (Figure 9). It was suggested that the sulfur atom is stabilizing the molecule on the metal surface, while the methylene groups induce the chiral arrangement of **17** on the Au(111) surfaces. The chirality is attributed to the methylene spacers of the anchoring legs, which are a slightly mismatched between neighboring molecules and formed both clockwise and counter-clockwise pinwheels in the chiral tripods. The surface-induced chirality in a self-assembled monolayer of **17** was confirmed by STM measurements and both possible mirror configurations were observed. It turned out, that the surface chirality is strongly dependent on the surface coverage of the substrate. The formation of the racemic mixture was observed at low surface coverages, while at higher surface coverages, the racemic form was converted into the enantiomerically pure segments, which was assisted by a thermally activated diffusion process. In this study it was shown for the first time that achiral molecules may form 2D homochiral arrays on solid surfaces.

**Figure 10:**
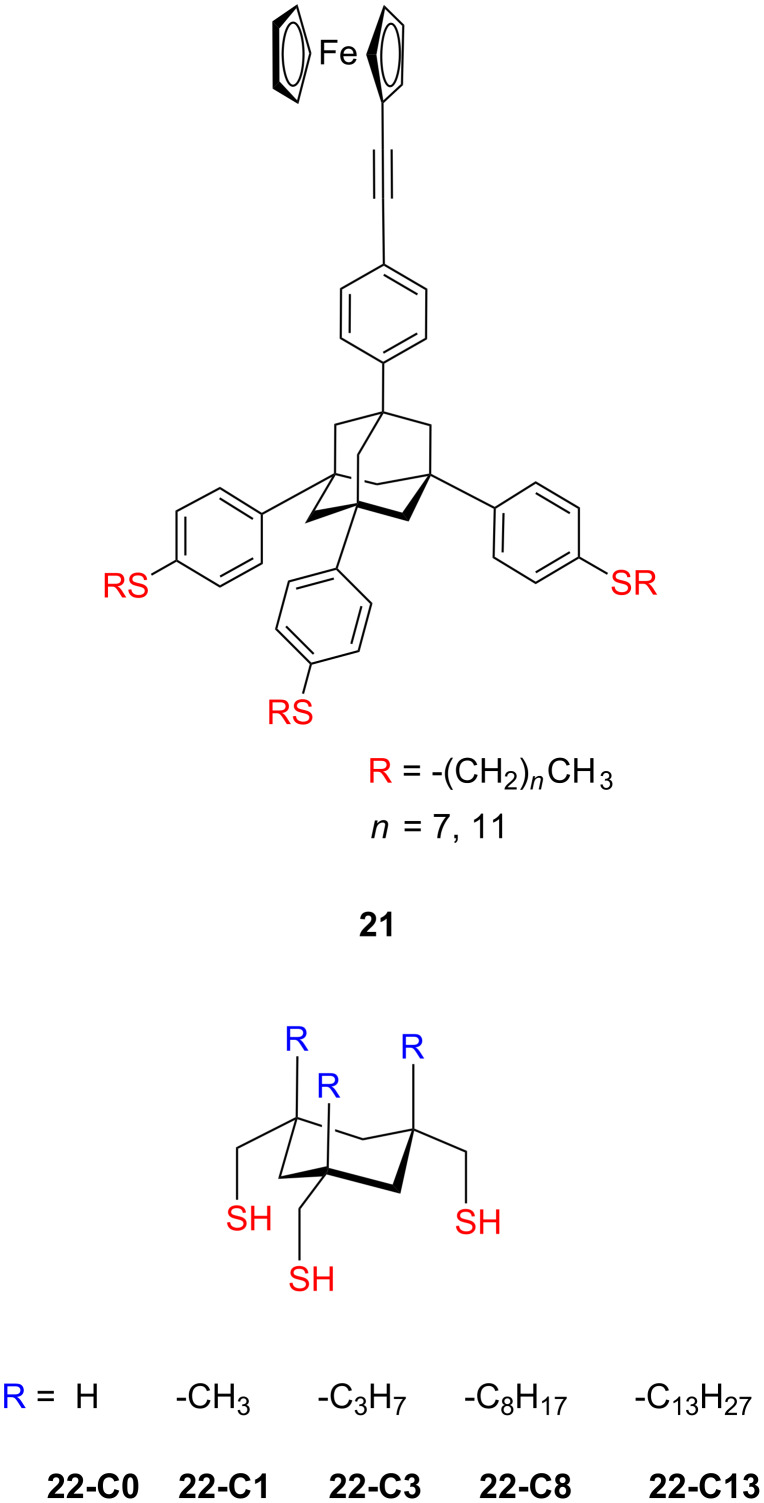
Adamantane and cyclohexane-based tridental platforms.

Kitagawa et al. also recently studied adamantanes terminated with ferrocenyl **19** [87,108–109] and 2,2’-bithiophene **20** [110] groups on gold surfaces (Figure 7). Molecular tripods with perpendicular ferrocenyl groups formed a well-ordered, tight electroactive SAM, where all three thiols were chemisorbed on the gold substrate, which was confirmed by PM-IRRAS and XPS analyses. Reductive desorption of chemisorbed molecules from Au(111) revealed a high surface coverage of adsorbed molecules through three anchoring points and the obtained surface density is in agreement with the value determined by the STM analysis. The very small value of anodic to cathodic peak separation (Δ*E*_pp_ = 7 ± 1 mV) and the full width at half maximum (Δ*E*_fwhm_ = 93 mV) fits almost perfectly with the predicted values for an ideal Nernstian system (Δ*E*_pp_ = 0 mV, Δ*E*_fwhm_ = 3.53*RT*/*nF* = 90.6 mV at 25 °C and *n* = 1) [111], corroborating that the lateral interaction between neighboring redox active ferrocene units is negligible. The bulkiness of the adamantane platform, which is higher than the volume of ferrocene, leads to a spatial arrangement with laterally separated ferrocenyl tail groups protruding from a surface. Consequently, no further dilution of the molecules is required. The rod-like substituent is almost perpendicular to the plane determined by the three thiols and the head ferrocenyl group is 16 Å above the gold surface, as optimized by density functional theory (DFT) calculations. The extended analysis of the STM image revealed that the SAM structure of ferrocenyl adamantane **19** exhibits the same ordering and stability as that of 1-bromo-3,5,7-tris(sulfanylmethyl)adamantane **17** [108], where the SAMs are stable under low-bias-voltage scanning, i.e., with a sample bias voltage lower than 1 V. The STS measurements confirmed the characteristic molecular resonance states (HOMO−1, HOMO and LUMO) originating from the ferrocene group when spectrum was measured at ferrocenyl adamantane **19**. The STS mapping succeeded in imaging the spatial distribution of the HOMO state of ferrocenyl adamantane **19**, which is localized at off-center of the adamantane-core frame. 2,2’-Bithiophene terminated dyads **20** [110], formed also SAMs on Au (111) trough three-point adsorption, but due to the *anti–syn* conformational flexibility of the perpendicular 2,2’-bithiophene rod like structure, the surface coverage was lower than that observed for the ferrocene-terminated adamantane **19**. This is in contrast to the fact that *n*-alkanethiols form closely packed SAMs, in which flexible alkyl chains are fixed at a linear conformation to maximize the intermolecular affinity. Recently Weidner and co-workers presented a study of adamantane-based larger tridentate ligands **21** comprising three long alkylsulfanyl chains (C8, C12) and a redox-active ferrocenyl tail group (Figure 10) for the preparation of redox-active SAMs on Au(111) substrates [89]. These tripodal molecules **21** form almost homogeneous, well-ordered, and fairly densely packed SAMs according to the XPS, NEXAFS spectroscopy, and sum frequency generation (SFG) spectroscopy measurements. Also, the calculated thickness based on the XPS data is in agreement with monolayer coverage. The perpendicular orientation and scope of spatial alignment for different alkyl chains exhibit that lateral interactions between neighboring molecules via the long-chain anchoring groups play an important role for the surface assembly. Tripodal platforms bearing shorter octylsulfanyl tentacles provided a lower packing density and film order than the ones with longer dodecylsulfanyl chains. The fact that the chain length of alkyl legs is crucial for the molecular self-assembly indicates that the driving force for the surface arrangement is based on lateral van der Waals interactions of neighboring alkyl chains, similar to alkane thiols on gold. These results suggest that employing of long-chain alkylsulfanyl groups can minimize the steric hindrance between bulky tail groups and show a possible pathway to get well-ordered tripodal scaffolds arranged on metal surfaces. A similar behavior was also recently observed in SAMs of flat platforms based on triazatriangulenium (TATA) cations [98]. Also a tripodal system based on an adamantane core unit with acetyl protected thiol anchoring groups and an azobenzene head-group was reported for the preparation of photochromic SAMs on gold surfaces [112].

Another type of rigid platform, based on the cyclohexyl-based tridental platform **22-C*n*** (Figure 10), was pioneered by Lee and co-workers [113–114]. First they synthetized two tridentate alkane thiols **22-C0**, and **22-C1** with cyclohexyl head-groups and used them to prepare SAMs on gold [113]. XPS measurements revealed that while the adsorption of **22-C0** led to multiplayer films containing oxidized sulfur species (e.g., disulfides and sulfones), the adsorption of **22-C1** lead to monolayer films with ≈90% of the sulfur atoms bound to gold, and no oxidized sulfur species were observed. This behavior is attributed to the presence of the methyl groups in **22-C1**, which stabilized the cyclohexane conformation and enhanced the chemisorption of adsorbed molecules. Ellipsometric measurements and analysis by XPS indicates that the thickness of SAMs formed from **22-C1** is about 5 Å, which is consistent with its molecular dimensions calculated by molecular modelling assuming a planar conformation. In contrast, **22-C0,** which formed multilayers, yielded films with a thickness ranging from 11 to 15 Å. In an extended study, they prepared and studied five tridentate platforms **22-Cn** (*n* = 0, 1, 3, 8, 13) having different upward alkyl chain lengths, where the cyclohexane ring serves as the platform between three alkyl tail groups and the three thiol-containing head groups [114]. Ellipsometric measurements of cyclohexyl-based tridental platforms exhibit that the thickness of these SAMs is significantly diminished as compared to alkylthiol SAMs of corresponding chain lengths. The conformational order in these SAMs as determined by the contact angle measurements and the PM-IRRAS spectra indicate an overall decreasing trend as follows: C_18_H_37_SH >> **22-C13** > **22-C8** > **22-C3**. XPS measurements revealed that the sulfur atoms of these alkylated platforms are attached to the gold surface, and perpendicular alkyl tail groups are loosely packed, compared to normal alkylthiol SAMs. Nevertheless, the concentration of absorbed molecules as revealed by XPS measurements corresponds with the model where the anchoring groups are closely packed on the surface with a parallel arrangement of cyclohexyl ring in the layer and with upwards alkyl substituents protruding from the surface. Moreover, the XPS analysis of the thermal stability of SAMs confirmed that the monolayer of the cyclohexyl platform with the longest alkyl chain **22-C13**, is significantly more stable than the corresponding octadecylthiol SAM, which is attributed both to the strong lateral van der Waals interactions between the long alkyl chains and to the chelate effect of the tripodal scaffolds.

### Aliphatic multipodal adsorbates

**Figure 11:**
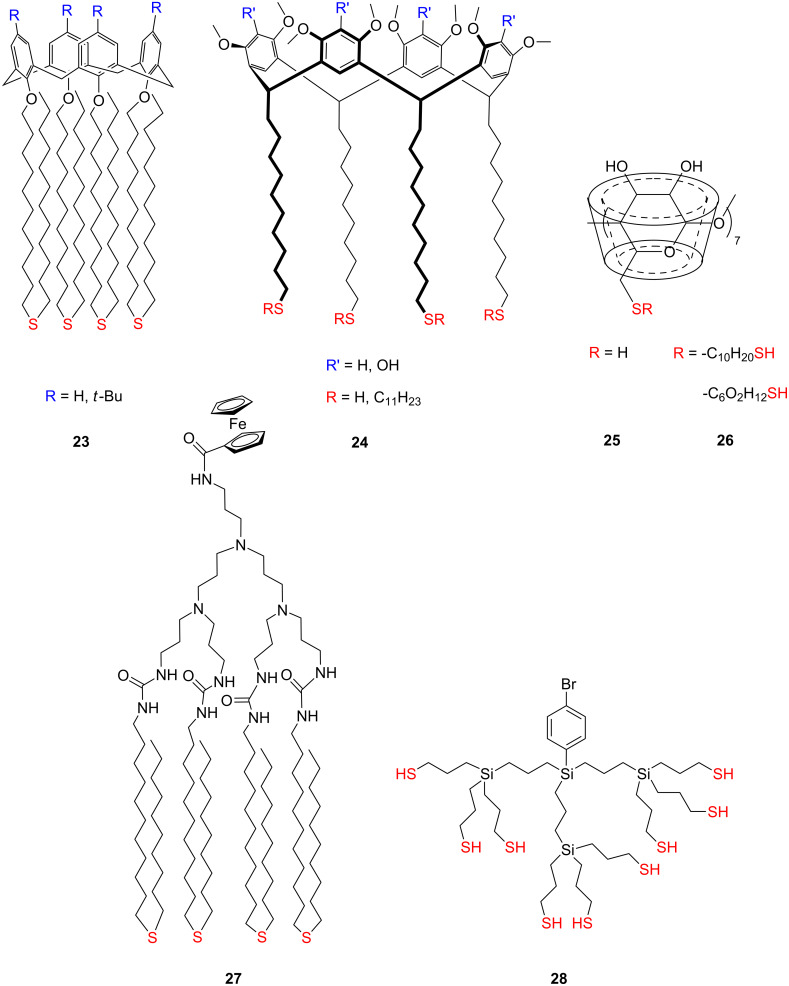
Structure of aliphatic multipodal adsorbates.

Several types of aliphatic multidental platforms (Figure 11) on gold have been reported, including calix[4]arene-based **23** [115], resorcin[4]arene-based **24** [116–118], ß-cyclodextrin-based [119–123] thiols **25** and sufides **26**, as well as other thiol-terminated dendrimers **27**–**28** [104,124]. Although this multipodal approach with several anchoring points enhanced the stability of some of these platforms on gold, the self-assembled monolayers are typically poorly ordered due to the presence of long alkyl chains as revealed by IR spectroscopy analysis of ß-cyclodextrin and resorcin[4]arene scaffolds. Substitution of these platforms with long dialkylsulfides with a lower affinity to gold improved the lateral mobility of monolayers. The increased mobility of the molecules and the influence of the additional alkyl chains led to an increase of surface coverage, and improve the self-assembly process. But these long alkyl chains are forming an insulating adlayer that reduces the electron transport between the metal electrodes, what diminish their applications in the molecular electronics. Therefore, finding a compromise between a proper geometry of multipodal molecules without simultaneous hindering the electrical properties remains a scientific challenge. To circumvent this behavior, several research groups utilized rigid conjugated platforms based on aromatic systems to make a strong and more conductive contact.

### Aromatic tripodal adsorbates

**Figure 12:**
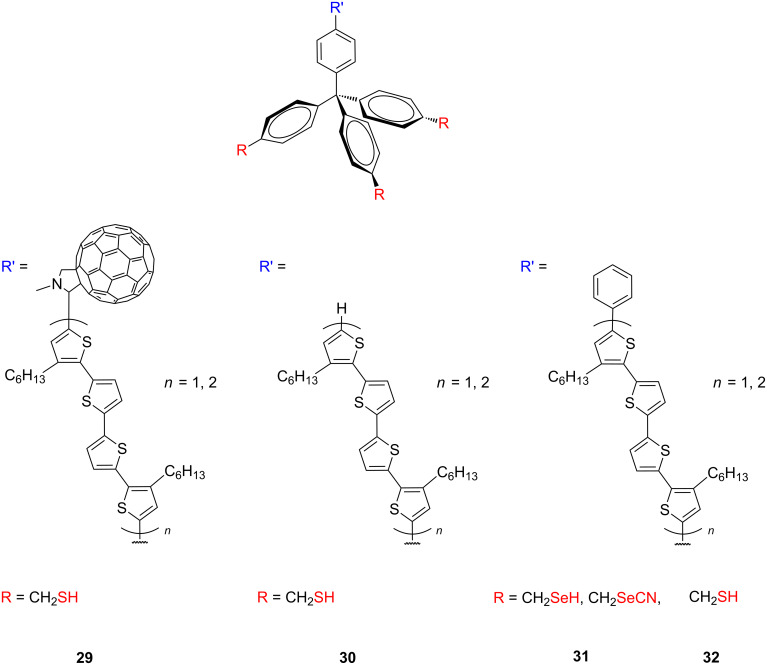
Functionalized tetraphenylmethane tripods **29–32**.

The synthesis of tetraphenylmethane-based anchor with three sulfanylmethyl feet was pioneered by Aso and co-workers [92]. They designed and studied [60]fullerene-linked oligothiophene tetramer and octamer derivatives bearing a tripodal rigid anchor **29** (Figure 12), allowing such molecules to form a stable and well-defined arrangement of molecules on the metal surfaces for the further construction of highly efficient molecular photovoltaic devices. They corroborated significant influence of the rigid tripodal anchor to stabilize molecules on the surface by photoelectrochemical measurements, where the molecules comprising a tetraphenylmethane anchoring platform show a significantly higher photocurrent density than the same system with a monopodal anchoring group. This can be attributed to a stable arrangement of a well-decoupled oligothiophene chromophore on the gold electrode, which suppresses quenching of the excited states of the chromophoric unit both by lateral interactions between neighboring molecules in the monolayer and by the gold electrode.

Further electrochemical studies of SAMs of two tripodal oligothiphene-bearing thiols **30** (Figure 12) on Au(111) indicated that the packing within the SAM of shorter oligothiophene-terminated tripods (*n* = 1) is more compact than that of the longer ones [125]. In the following series of tower-shaped molecules there are two factors that significantly lower molecular order in monolayers on gold surfaces, the increasing length of rod-like oligothiophene moieties standing upwards on the surface as well as the higher number of hexyl side chains along the structure. Consequently these features were identified as unfavorable factors for charge transport through the SAM. In the case of the shorter oligothiophene tail in **29**, the π-conjugated tail has an appropriate length, allowing for compact packing of the molecules. A structural feature reflected in a greatly enhanced charge transport through the SAM. They demonstrated the fabrication of organic light-emitting diodes (OLEDs) where the gold surface of the anode was coated with a self-assembled monolayer. In contrast to the bare gold device, the device consisting of the gold electrode coated with tripodal oligothiophene monolayers exhibited a remarkably improved electroluminescence performance, which lead to a significantly reduced operating voltage of the corresponding OLED, resulting in high quantum efficiency, better stability, higher maximum brightness, offering reduced resistance, and permitting higher current densities for a given bias voltage. Aso and co-workers have also synthetized selenium-terminated tetraphenylmethane tripods **31** bearing three selenocyanate or selenol arms as anchoring groups (Figure 12). CV, XPS and ultraviolet photoemission spectroscopy (UPS) measurements of their monolayers on a gold surface were investigated and the results were compared with those obtained from the thiol-terminated analogues **32** (Figure 12) [39]. They found that all three selenol groups of the tripod are bound to gold surface and the selenol monolayer is electrochemically more stable than that with thiols. Furthermore, a comparative UPS study of the gold–thiol and gold–selenol tripodal interfaces showed that the charge-injection barrier between the Fermi level of the gold electrode and a single discrete energy level of the tripodal molecule at the electrode–molecule interface was smaller in the gold–selenium tripodal interface. This lower barrier for selenol-terminated tripods in the gold–selenium interface leads to a low resistance when small voltage biases are applied, which makes selenol a better anchoring group with a well-defined electronic coupling and faster electron transport to gold electrodes than thiol for further elaboration toward single-molecule devices. These results are consistent with the trends reported recently [38].

**Figure 13:**
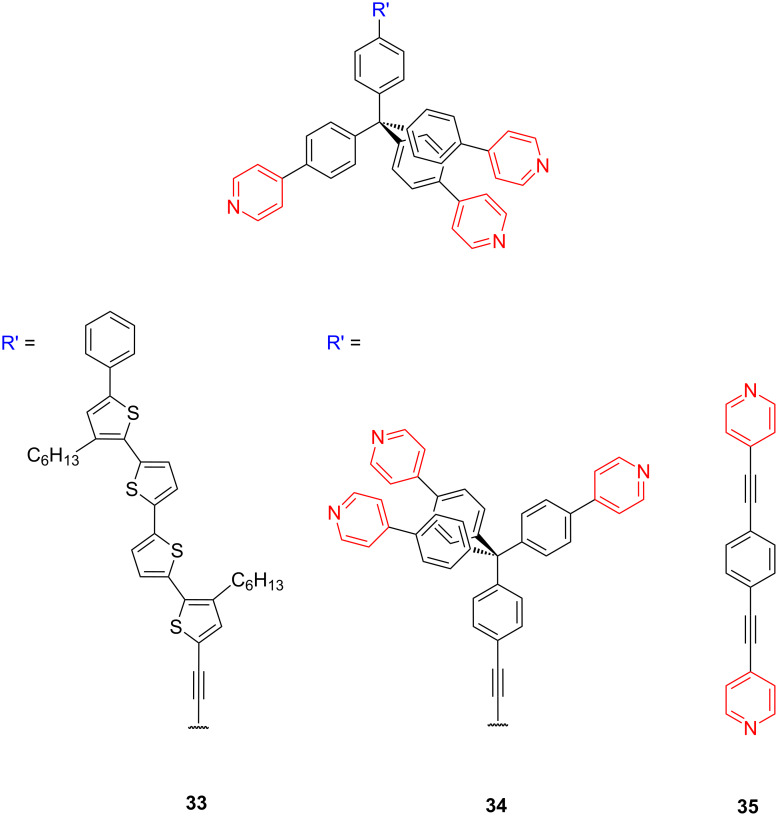
Structure of pyridine terminated platforms **33–35**.

Aso and co-workers also recently designed and synthesized the [4-(4-pyridyl)phenyl]methyl tripodal platforms **33** and **34** (Figure 13) to realize robust single-molecule junction with a gold electrode and to achieve effective hybridization of the pyridine π orbitals with the gold electrode [35].

**Figure 14:**
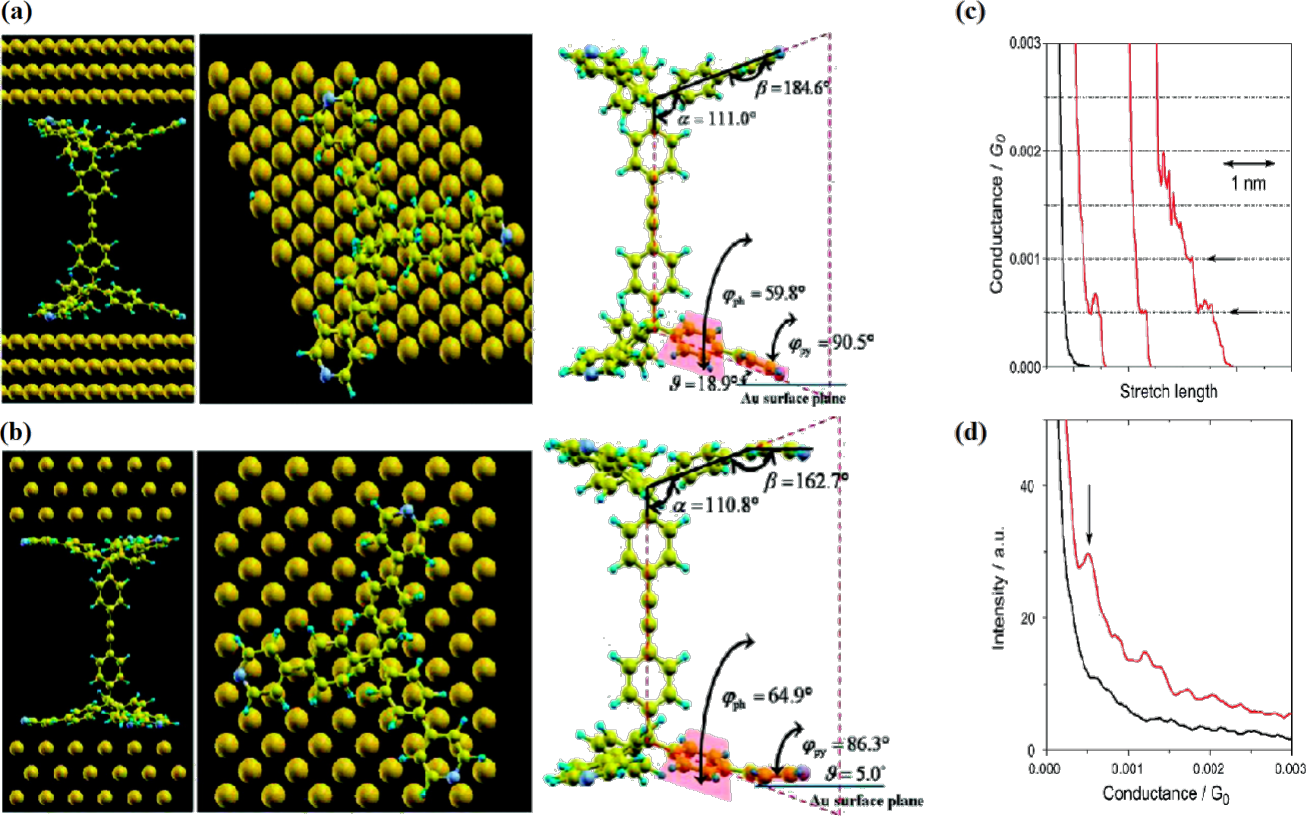
Structures of the junctions used for the ab initio transport calculations, (a) **34** (111) model and (b) **34** (001) model. The left panels show side views of each system, whereas the middle panels show views from the top. The right panels give the details of the parameters to identify the conformation for **34** platform, such as dihedral and bending angles. (c) Conductance traces measured when breaking the Au point contacts in solutions with (red) and without (black) **34**. (d) Corresponding conductance histograms constructed without data selection from 1000 traces. Each histogram is normalized by the number of traces used to construct the histogram. The bin size is 10^−5^*G*_0_. Reprinted with permission from [35], copyright 2011 American Chemical Society.

SAMs of 4-pyridyl-terminated tripodal **33** as well as monopodal platforms bearing both a redox-active oligothiophene tail group for CVs, and a (triisopropylsilyl)ethynyl tail group for XPS measurements were evaluated. CVs of the redox-active tripodally modified gold electrodes displayed a reversible one-electron redox wave at a surface coverage of 7.1 × 10^−11^ mol·cm^−2^. Furthermore, they examined the electrochemical stability of self-assembled monolayers on gold electrodes and found that 30% of the tripodal molecules still remain on the surface after 10 scans within the range of 0–0.55 V. XPS measurements revealed that the π orbitals of the pyridines contributed to the physisorption of the tripodal platform on gold. Measurements of single-molecule conductance were successfully carried out using modified STM techniques for single-molecule junctions that consisted of the tripodal anchors and a diphenyl acetylene linker **34**. The single-molecule conductance of a metal–molecule–metal junction based on the pyridine-terminated tripodal structure **34** exhibited conductance values of (5 ± 1 × 10^−4^*G*_0_), about two orders of magnitude higher than that of the previously reported monopodal pyridine analogue **35** (3.5 × 10^−6^*G*_0_, Figure 14c,d) [126]. Ab initio charge-transport calculations through the molecular junction based on pyridine-terminated tripodal platforms **34** fully matched with the experimental results and revealed that the electron-deficient π* orbitals of the pyridine anchor subunits directly interact with the gold electrode and result in a robust molecular junction via the three pyridine units, where the LUMO dominates the electron transport via π-channel hybridization (Figure 14a,b).

**Figure 15:**
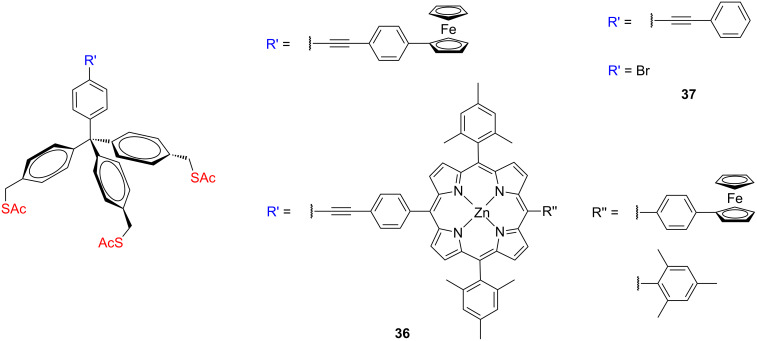
Redox-active tripodal structures **36** and **37**.

Lindsey, Bocian and co-workers synthetized several redox-active molecules bearing a tether composed of a tripodal tetraphenylmethane with three acetylsulfanylmethyl groups **36** (Figure 15) for surface attachment to examine the effects of spatial arrangement of the molecular structure on charge storage in SAMs [72]. The redox-active molecules include ferrocene, zinc porphyrins, magnesium phthalocyanine, and triple-decker lanthanide sandwich complexes.

They studied the electrochemical behavior and stability of these redox-active molecules both in solution and in SAMs on gold electrodes and found that employing the sulfanylated tripodal platform significantly enhanced the stability and the lateral order of the molecules on gold surfaces as compared with the corresponding monopodal anchor groups. However, the electron-transfer and charge-dissipation characteristics of the tripodal thiolated molecules and monopodal thiolated species are generally similar, which proved that the redox-active termini are electronically well decoupled from the metal surfaces. These two important features demonstrate the ability to attach these redox-active molecules to the metal surfaces via a stable acetylsulfanylmethyl terminated tripodal scaffolds as a step towards molecular-based information storage devices. SAMs of two tripodal thiol-terminated metalloporphyrins **36** (Zn and Cu) and three benchmark tripods were further studied by XPS and FTIR measurements on gold substrates [73]. The benchmark molecules **37** (Figure 15) include (1) two tripods containing a bromine atom at the perpendicular position of the apical phenyl ring and sulfanylmethyl or acetylsulfanylmethyl feet, (2) an acetylsulfanylmethylated tripod terminated in the perpendicular position with a phenylethylene unit. However, the results from the spectroscopic studies of these five tripodal derivatives **36** and **37** showed that none of the tripods attach to the gold surface via all three sulfur moieties. The average value of anchor thiols is in the range of 1.5 to 2. This nonuniformity of binding through the different SAMs might arise from steric interaction between co-deposited molecules. They also observed the similar surface coverage for both the *S*-acetyl-protected and free-thiol-terminated tripodal molecules on gold, which indicated that the effect of the thiol protecting group on the binding is negligible and the protecting groups are cleaved in situ during deposition. Furthermore, the binding features of these molecules are also independent of the chosen solvent, temperature, or deposition time as determined by IR analysis.

**Figure 16:**
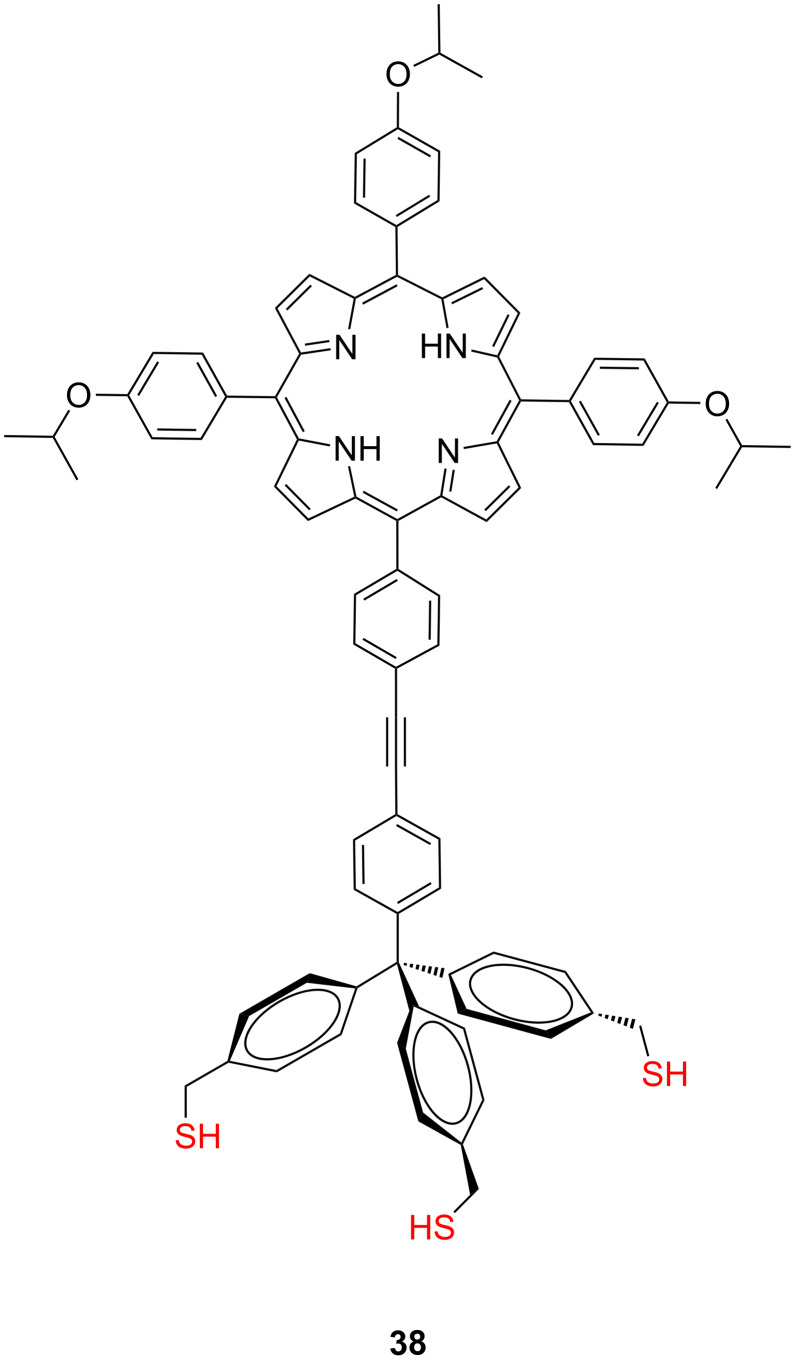
Self-decoupled porphyrin with a tetraphenylmethane scaffold **38**.

**Figure 17:**
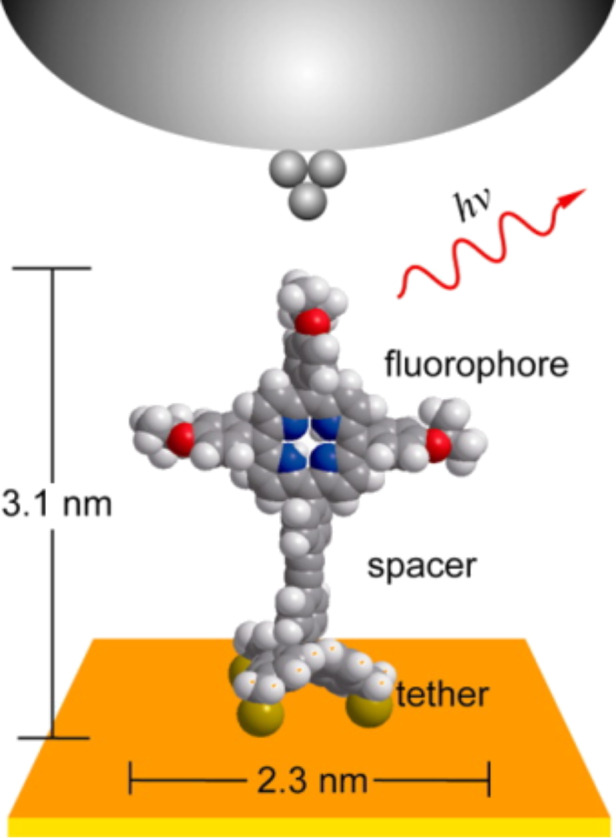
Schematic configuration of **38** on Au(111) and localized electrical excitation from a nanotip. Reprinted with permission from [74], copyright 2013 American Chemical Society.

Recently, Dong and co-workers have synthetized a self-decoupled porphyrin with a tetraphenylmethane tripodal anchor **38** (Figure 16) and deposited it on Au(111) using different wet-chemistry methods in order to assemble a single molecule electroluminescence STM experiment (Figure 17) [74]. The rigid tripodal anchor in this molecule not only acts as a robust decoupling spacer but also controls the orientation of the porphyrin molecule in the desired up-right standing position along the tip axial direction.

STM images revealed the formation of dispersed bright spots (ca. 3–5 nm), fitted to the single or aggregated molecule, placed perpendicularly to the Au(111) surface. This STM-junction operating in the tunneling regime was irradiated with a short excitation pulse to measure the molecular electroluminescence when excited by local electron tunneling. Electroluminescence from the excited molecules is a strongly unipolar process and depends on the polarity of the applied bias as revealed by STML spectra, which displayed electroluminescence exclusively at positive bias polarity (ca. 1.9 V). They attributed this unipolar behavior to both, the energy alignment determined by the position of the frontier molecular orbitals to the Fermi level of gold at the molecular interface and to the molecular tip–molecule–gold junction asymmetry. Based on these results, it was suggested that at a positive tunneling bias, a photo-excited hot electron from the STM tip resonantly tunnels into an excited state of the porphyrin molecule **38** strongly bounded to the gold surface, and the excited molecule then decays radiatively back to the ground state, which leads to a plasmon-enhanced electroluminescence of the single molecule in the STM junction. These results are of interest as fundamental studies of electrically driven single-molecule light sources that help to analyze and improve the mechanisms in molecule–electrode junction in organic light-emitting diodes.

**Figure 18:**
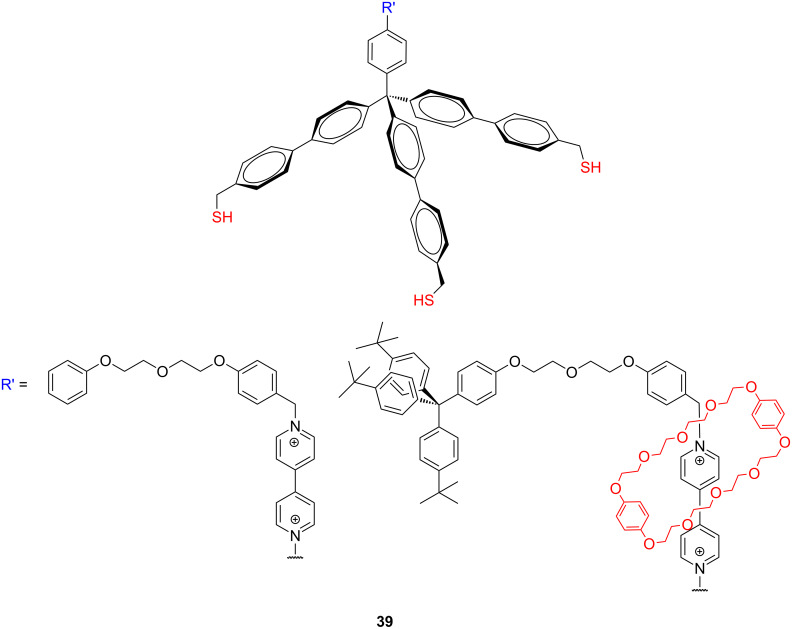
Structure of tripodal [2]rotaxanes **39**.

A tetraphenylmethane-based tripodal platform was also employed to immobilize oligonucleotide probes perpendicularly to the gold surface of DNA chips [127]. In order realize reliable DNA arrays for a reproducible, inexpensive, and high-throughput detection system for genetic analyses in clinical diagnostics, particular attention must be paid to form stable molecules with precise control over the spatial arrangement of oligonucleotide probes immobilized on a surface. Moreover, electrochemically controlled and potentially switchable tripodal [2]rotaxanes incorporating a viologene moiety, a crown ether, and sulfanylmethyl-terminated extended tetraphenylmethane anchoring group **39** (Figure 18) have been prepared and their SAMs on gold have been studied by cyclic voltammetry [93]. The thiol-terminated tripodal viologens formed oriented SAMs on the gold surface, and threaded crown ethers to form a hetero [2]rotaxanes with a surface coverage in the range from 10^−10^ to 10^−11^ mol·cm^−2^.

**Figure 19:**
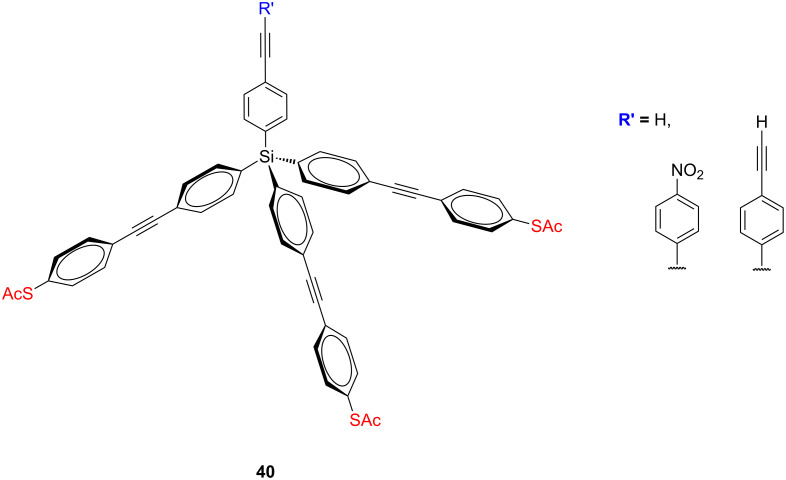
Tetraphenylsilane-based platforms **40**.

Analogously to the tetraphenylmethane tripods, also synthetically easily accessible tetraphenylsilane derivatives have been employed as rigid molecular scaffolds for the metal surfaces. But also in these molecular tripods, remaining sp^3^-hybridized silicon core atom leads to the electronic decoupling of molecules from a metal substrate. The synthesis of tetraphenylsilane-based tripodal platforms **40** (Figure 19) with three 4-(acetylsulfanyl)phenyl anchoring groups for chemisorption on gold and one sharp arm to act as a probe tip was pioneered by Tour and co-workers [75].

They firstly prepared the precisely defined molecular tripods that may act as scanning probe microscopy (SPM) tips. The silicon core in these tripodal platforms provides a suitable template for the required construction of the three legs and the probe arm, each length being variable using different rigid oligo(phenylene ethylene)s. However, self-assembly of these *para*-acetylsulfanyl-terminated tripods on a gold surface was inconsistent and molecules were tilted when attached to the gold surface. The ellipsometry thicknesses and surface IR studies suggested that two of the thiols would bind while the third projected off the surface. A structurally improved version of tetraphenylsilane-based platform bearing three sulfanylmethyl anchoring groups at *meta*-positions relative to the ethylene groups provided successful coupling to the metal surfaces via three sulfur bonds.

**Figure 20:**
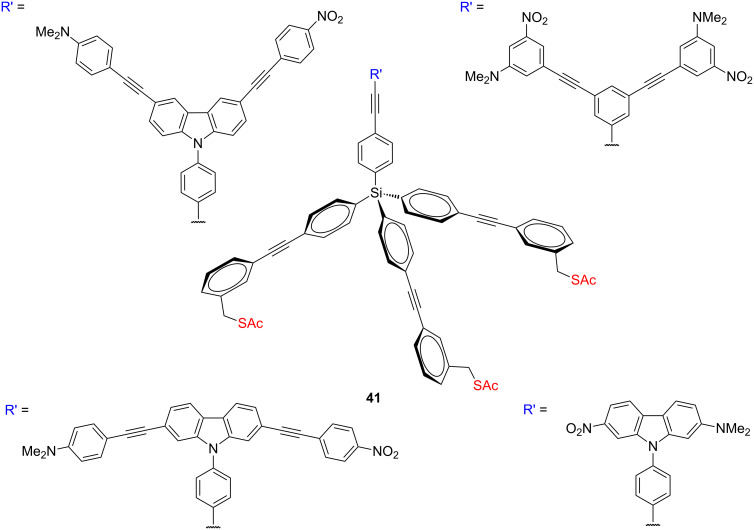
Structure of surface-bound molecular motors **41** for gold surfaces.

Several molecules **41** (Figure 20), containing a pair of electron donor–acceptor arms (carbazole core and OPEs with a strong dipole) and a tripodal base, that might be useful as surface-bound molecular motors have been synthetized [76]. The geometry of the tripod base allows the tripod to project upwards from the gold surface after self-assembly. The packing of these molecules on gold was investigated by using ellipsometry. These results showed that the thiol moieties on the legs of the molecular tripods allow them to form SAMs on gold, with molecular thicknesses that are consistent with the calculated molecular heights.

**Figure 21:**
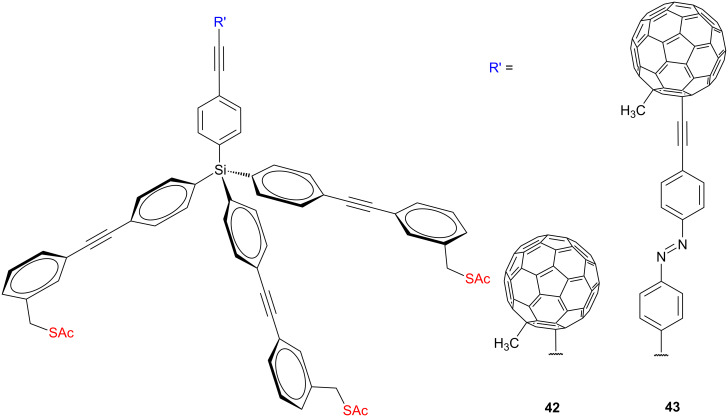
Fullerene-terminated tetraphenylmethane platforms **42** and **43**.

In an extended study of a series of fullerene-terminated molecules with different thiol and protected thiol alligator clips and platforms, the rigid tetraphenylsilane tripods **42**, **43** (Figure 21) were also employed to get better packing of molecules on the gold surfaces [77–78]. However, it was found that these molecules terminated with bulky fullerene moieties displayed a more complicated SAM-forming behavior on gold surfaces. Mainly multilayers and/or head-to-tail assemblies were observed instead of well-ordered monolayers, which was attributed to strong fullerene–fullerene and fullerene–gold interactions. This behavior of fullerene derivatives in SAMs on gold was revealed by several spectroscopic and electrochemical techniques including XPS, ellipsometry, and CV analysis. The cyclic voltammetry results confirm the noncrystalline, less defined liquid-like loose packing of the tripodal platforms bearing fullerene tail groups on gold surfaces. The SAMs were composed of complex mixture of several conformations with different numbers of covalently bound anchoring thiols. The XPS analysis of these fullerene SAMs showed a considerable amount (in the range of 40%) of sulfur atoms that were not bound to the surface. This hints at the limited control over the spatial arrangement of the molecules.

**Figure 22:**
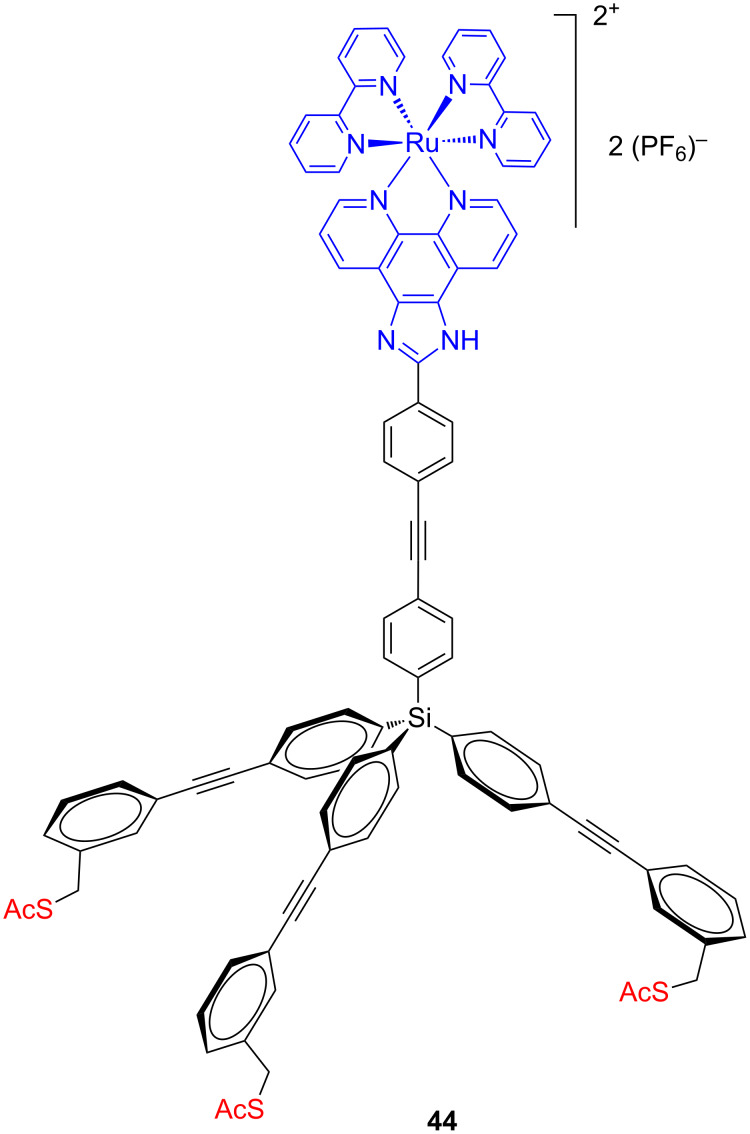
Structure of the tripodal luminescent ruthenium complex **44**.

The luminescent ruthenium complex **44** (Figure 22) containing an acetylsulfanylmethyl-terminated tetraphenylsilicon-based tripod linked through a rigid spacer to a phenanthroline derivative was synthetized and the photophysical and electrochemical behavior of the complex was studied in solution and on a gold surface [79].

The luminescent ruthenium complex consists of two bipyridine units and the phenanthroline ligand bearing a five-membered ring for its perpendicular mounting on the rigid tripodal platform. The authors argue that the benefit of this molecular design is not only the well-defined binding geometry due to the multipodal platform, but also the electronic decoupling of the luminescent ruthenium complex from the surface. However, the emission of the ruthenium complexes self-assembled on the surface was quenched by the gold surface and at least two out of the three sulfur anchoring groups surely are attached to the gold surface according to the XPS measurements, which is in analogy to previously reported model compounds based on the same tripodal subunit [76]. Furthermore, the excited-state lifetimes of these ruthenium-tripodal SAMs on gold were measured by using a time-resolved confocal microscope and the conductivity of these redox-active molecules in the molecular junction was investigated with gold and indium gallium eutectic electrodes. The results showed that the monolayers are extremely stable, densely packed because of the tripodal system and rectification behavior was observed.

**Figure 23:**
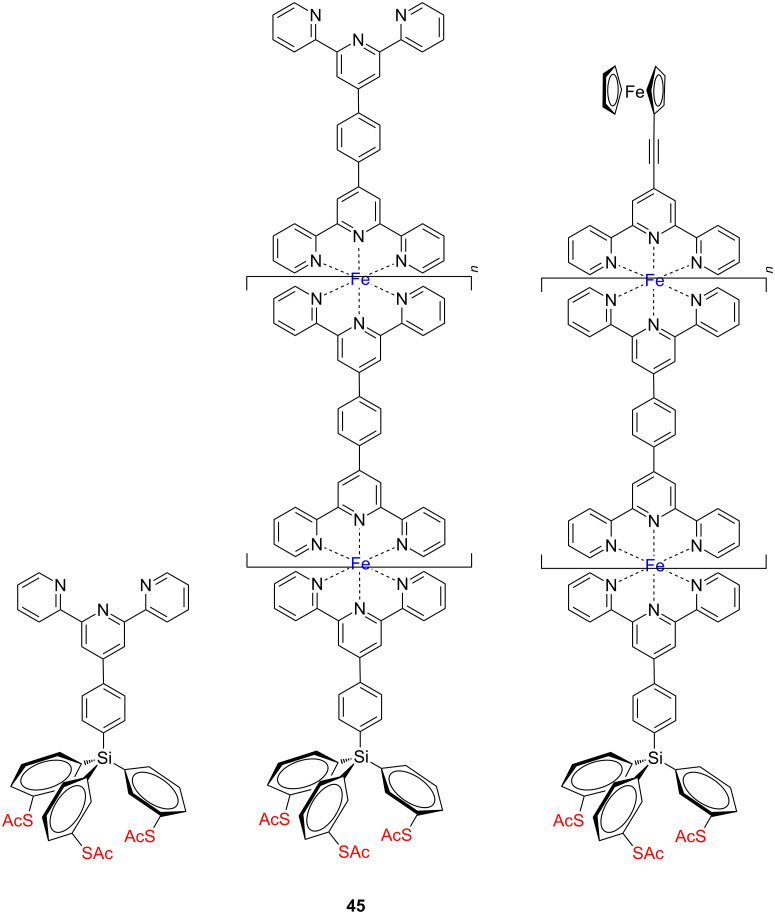
Bis(terpyridine)–Fe(II) oligomer wires terminated with a tripodal scaffold **45**.

Recently Nishihara and co-workers employed the tetraphenylsilane scaffold bearing in the *meta-*position acetylsulfanyl groups for the preparation of orthogonal bis(terpyridine)–Fe(II) oligomeric wires **45** on Au(111) (Figure 23) and measured the electron transfer through the oligomer wires [80]. This was the first example where acetylsulfanyl anchoring groups were directly bound to a π-conjugated tripodal platform.

The fast electron transfer in these structures should be also supported with the sp^3^-hybridized Si core, which is known to provide some σ–π conjugation with the aromatic substituents. We note that all tripodal adsorbates reported so far adopted non-π-conjugated aliphatic thiols as an anchoring group on the metal surfaces, such as sulfanylmethyladamantanes and sulfanylmethylphenyl. The formation of the SAMs of a tripodal terpyridine anchor ligand on gold was optimized to ensure that all sulfur atoms are chemisorbed, which was determined by XPS and IR measurements. The bottom-up fabrication of bis(terpyridine)–Fe(II) oligomer wires from the SAM of a tripodal terpyridine anchor ligand on gold proceeded quantitatively as determined by CV, and the perpendicular arrangement of molecular wires on a surface was corroborated by AFM and cross-sectional scanning electron microscopy (SEM). The obtained thickness of the film was in good agreement with the height estimated by molecular modelling. Finally, intrawire electron transport behavior was investigated and found that the tripodal scaffold realized fast electron transfer through the oligomeric wires, showing large *k*^0^_et_ values.

**Figure 24:**
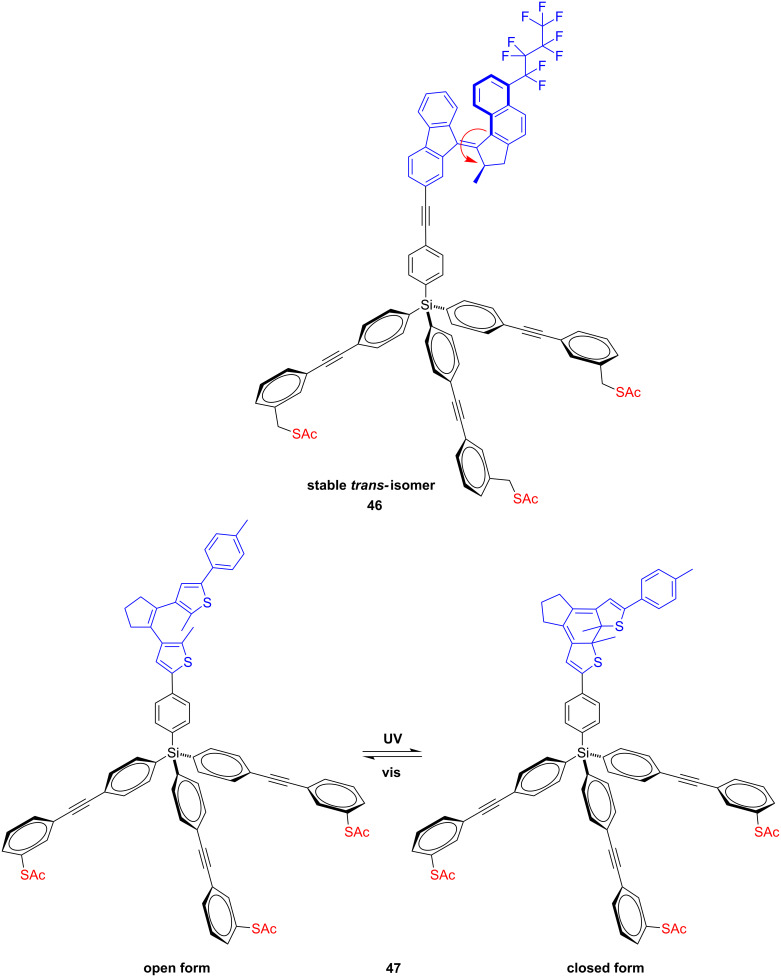
Structure of altitudinal light-driven molecular motors **46** and **47** for gold surfaces.

Ferringa and co-workers recently published a study about a light-triggered altitudinal molecular motor **46** (Figure 24) that contains as a stator a bulk tripodal platform terminated with three sulfanylmethyl anchors for final self-assembly on gold surfaces and as a rotor a photoresponsive molecule bearing a hydrophobic perfluorobutyl chain to change the surface wettability upon irradiation [94].

The SAMs of a tripodal molecular motor were characterized by XPS, UV–vis absorption spectroscopy and water contact angle measurements on flat gold surfaces. Selection of the bulky tripodal platform guaranteed an effective separation between the gold surface and the light-driven molecular motor, which was mounted to the tripod. As a consequence, quenching of the excited state by the gold surface does not effect the photoisomerization process of the central alkene axle. Contrary to the previously reported asymmetric altitudinal motors enriched by fluoro substituents that did not change the water contact angle under photo-irradiation [128], the current system changed the contact angle of a water drop by up to 16° upon irradiation. This was the first example exhibiting that the wettability of gold surfaces functionalized with light-driven molecular motors can be modulated by UV irradiation. In summary, the following multipodal approach is crucial for the future fabrication of functional nanoscale devices that can be used to exploit the rotary motion to perform mechanical work at the molecular level, to control intermolecular interactions on the surfaces and to measure the rotation and torque of a light-driven single-molecule motor on gold using single-molecule methods. In an additional study they reported a tripodal system for anchoring photochromic dithienylethylenes **47** (Figure 24) on a gold surface and showed that the tripodal dithienylethylenes forms stable monolayers on gold in which all three thiol legs are adsorbed to the surface as determined by CVs, surface-enhanced Raman spectroscopy (SERS) and XPS measurements [95]. These results were compared with solution studies, solid-state Raman spectroscopy, and density functional theory (DFT) calculations. The SAMs formed were found to be stable under the conditions applied for photochemical switching and, to a lesser extent, electrochemical switching. Furthermore, they demonstrated that **47** displays reversible photochemical and electrochemical switching, both in solution and on gold substrates. Importantly, although **47** exhibits photochemical switching fatigue in solution, this is not observed during photochemical switching of SAMs of **47** on gold surfaces.

**Figure 25:**
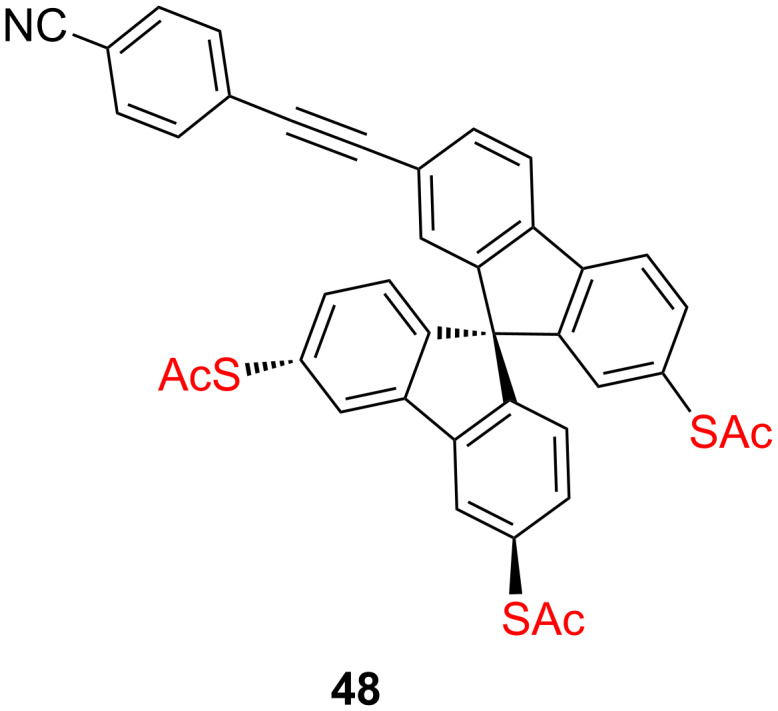
Structure of the rigid 9,9’-spirobifluorene platform **48**.

While several of these multipods allow for a perpendicular arrangement of rod-type molecular structures, the electronic coupling of the π-system of the rods to the metal states is limited due to the tripodal architectures comprising sp^3^-hybridized atoms. Our group recently developed a tripodal platform as modular anchoring subunit providing both, a vertical arrangement of the molecular rod and its electronic coupling to the gold substrate. A rigid three-dimensional 9,9’-spirobifluorene **48** (Figure 25) with acetylsulfanyl anchoring groups in the positions 2, 3’ and 6’ and a synthetically variable position 7 allowing us to introduce rigid-rod-type structures experiencing an efficient coupling to the metal electrode in a modular manner [129].

**Figure 26:**
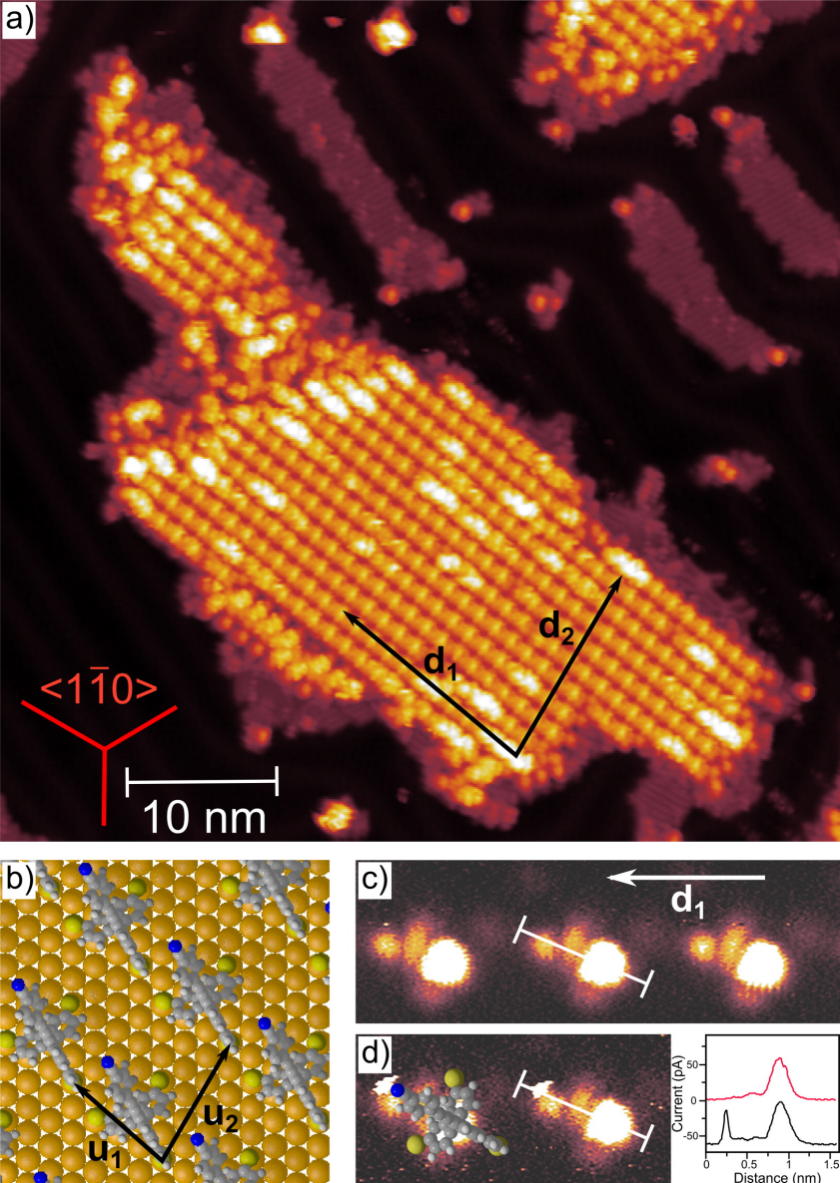
(a) Highly ordered island of molecular tripods **48** (yellow) and remaining CH_2_Cl_2_ (dark purple) on the Au(111) surface. (b) Unit cell of the molecular islands as extracted from the directions and distances in (a) with the molecular configuration as extracted from (c). (c) Constant-height mode image with submolecular resolution. (d) STM image of the same molecules as in (c) scanned at lower distance with a model of the molecule superimposed (model size is to scale). The inset cross sections show the distance between nitrile group and the spirobifluorene core. Reprinted with permission from [129], copyright 2014 American Chemical Society.

A first model platform **48**, comprising a *para*-cyanophenylethynyl as rigid-rod subunit in the position 7 was recently reported and displayed promising self-assembly features of this 9,9’-spirobifluorene platform on Au(111) and also corroborate the validity of the molecular design by a protruding rigid-rod molecular subunit, which was revealed by UHV-STM experiments (Figure 26). All together the quenching of the gold reconstruction, the commensurability with the surface structure and the orientation of the molecule as found by constant height imaging, support the concept of the rigid tripodal structure to stabilize the molecule on the Au(111) surface and to control the spatial arrangement of the molecular rod in an upright orientation.

**Figure 27:**
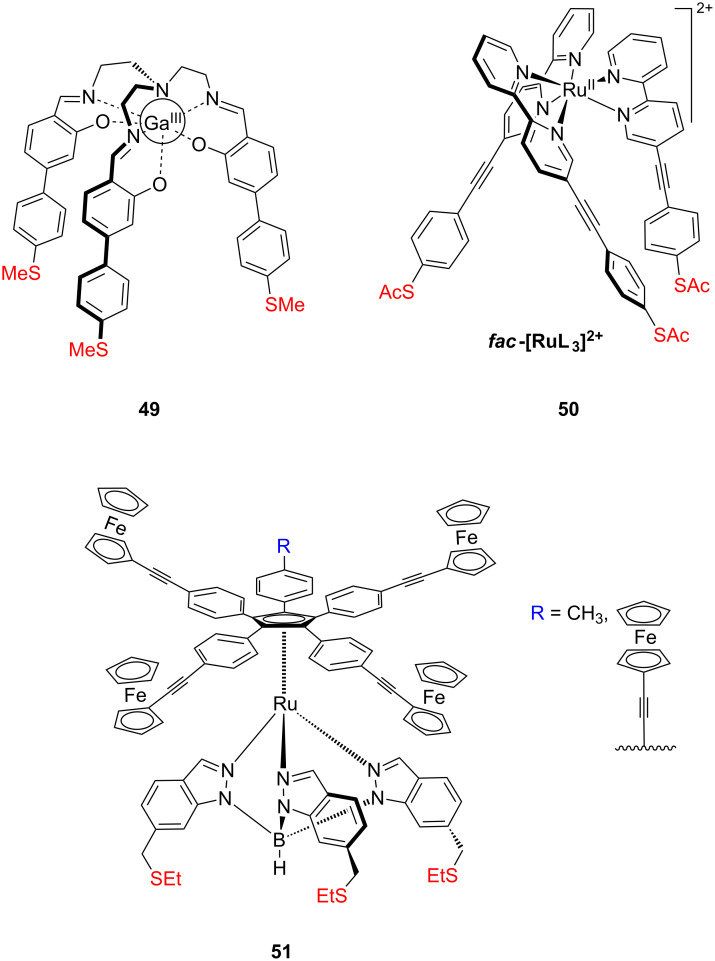
Organometallic tripodal scaffolds **49–51**.

We noted that also several organometallic complexes have been recently employed to serve as molecular platforms for the metal surfaces. Our group recently synthetized and employed a series of tripodal M(III) complexes (Figure 27) functionalized with three methylsulfanyl end groups for deposition on Au(111) [130]. The coordination core structure is based on a trensal [(tris(2,2’,2’’-salicylideneimino)triethylamine)] Schiff base ligand, which provides stable metal complexes. The Ga(III) complex **49** deposited on Au(111) was investigated in a UHV-STM and the experiments showed the tripodal shape of isolated molecules on the surface. However, the molecules most frequently lie on the surface and are rather attached via two legs while the third leg protrudes outwards. This arrangement probably arises from a strong interaction of the side and head part of the molecule with the Au(111) and also due to the presence of methylsulfanyl anchoring groups, which are exhibiting a lower affinity to the metal surfaces. In a further study, the conductance behavior of the feet of these tripodal structures with respect to their position and coupling to the surface electrode with the submolecular resolution of a STM was investigated and the results were supported by calculations of the electronic structure simulating the conformation of the molecule on the surface by DFT with dispersion corrections [131].

Also tripodal facial and meridional Ru(II) complexes **50** (Figure 27) comprising three conjugated legs with acetylsulfanyl anchoring groups were synthetized and isolated by our group [132]. Molecules of the facial Ru(II) isomer were deposited on Au(111) and studied in a UHV-STM. In contrast to the previously reported Ga(III) complex **49**, the *fac*-Ru(II) complex formed islands of dimers which exhibit a medium range order. Another organometallic tripodal platform was synthetized by Launay, Rapenne and co-workers and mounted on Au(111) surface [96,133–134]. They designed, and investigated by UHV-STM measurements, an azimuthal molecular rotor **51** (Figure 27) consisting of a five-arm rotor (penta-substituted cyclopentadienyl ruthenium(II) complex) on a molecular tripodal stator (tris(indazoyl)borate)-terminated with ethylsulfanylethyl anchoring groups. The motion and rotation mechanism of surface-bound molecular rotors was examined through STM analysis, and they showed that an azimuthal rotor adsorbed on gold can be rotated unidirectionaly in both clockwise and anticlockwise direction by selective exciting of different ferrocene arms of the upper rotator unit [96].

### Aromatic multipodal adsorbates

**Figure 28:**
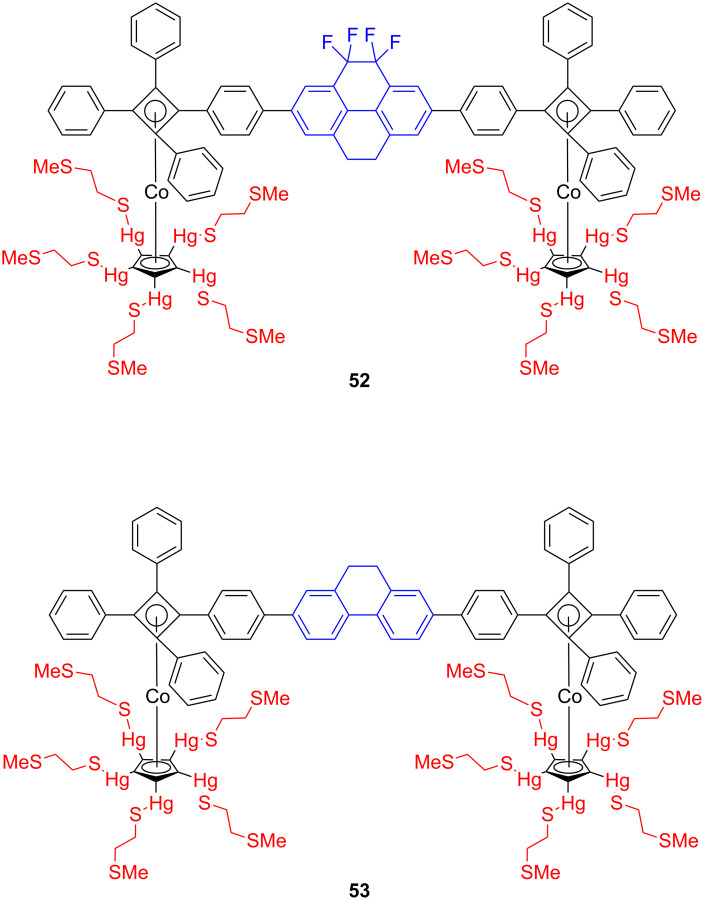
Dipolar and nonpolar altitudinal molecular rotors **52** and **53**.

The flat multipodal platforms have been also used for the mounting of molecules to the metal surfaces. For example, Michl and co-workers described the preparation of a compound whose molecules consist of two cyclobutadienecyclopentadienylcobalt sandwich stands carrying ten sulfur-containing “tentacles” with affinity to metal surfaces and holding an axle that carries a dipolar **52** (fluorinated phenanthrene derivative) or a non-polar **53** (pyrene derivative) rotator and served as a dipolar and nonpolar altitudinal molecular rotors (Figure 28) on Au(111) [97].

**Figure 29:**
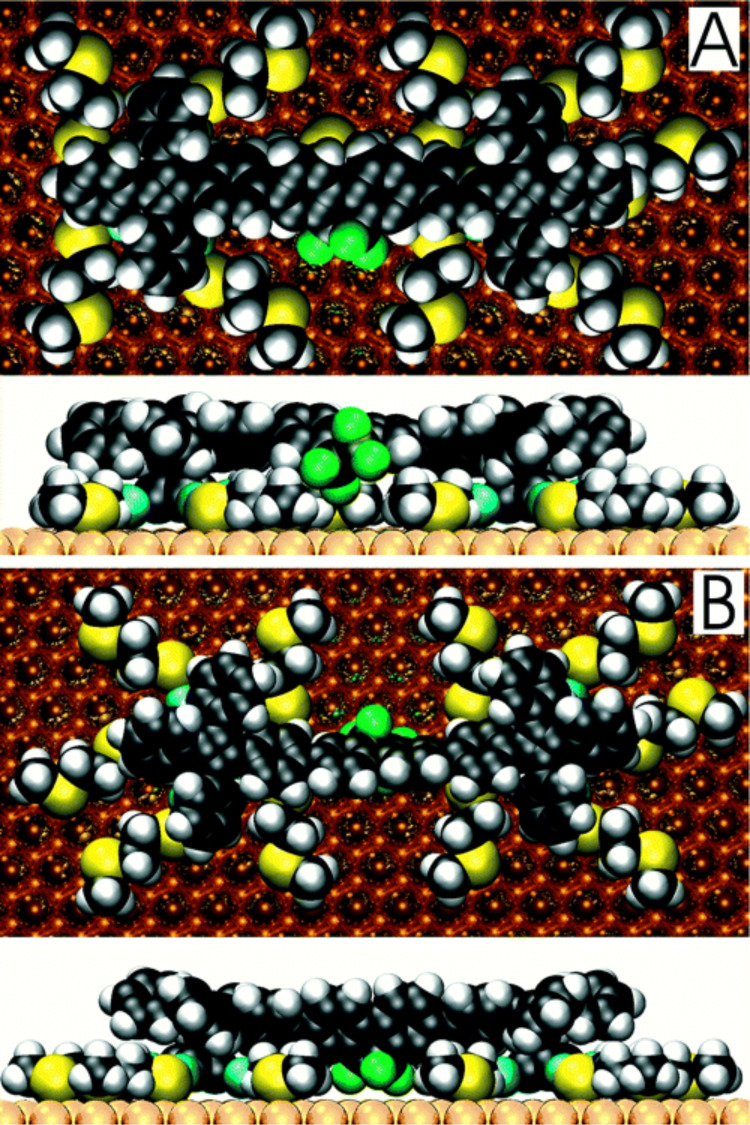
Optimized representative eclipsed (A) and staggered (B) conformations of the MMP diastereomer of **52** on Au(111). Reprinted with permission from [97], copyright 2004 American Chemical Society.

They fabricated monolayers and submonolayers on gold and this surface attachment of altitudinal molecular rotors provided with ten –HgSCH_2_CH_2_SCH_3_ “tentacles” has been monitored with ellipsometry, STM, and XPS spectroscopy [135]. These results all indicate that rotors indeed attach to a gold surface, with the rotor axle parallel to the surface, and without any inclination for multilayer growth, which is in agreement with the results of an IR study [136]. The STM analysis reveals that molecules organize on a gold surface and cover an area of about 2–3 by 4–5 nm^2^ per molecule. This value is in good agreement with the calculated one about 9 nm^2^ obtained for the expected conformation, where all ten sulfur-containing tentacles are attached to the surface. This value of the surface area also fits well with the footprint size of 8.5 nm^2^ per molecule obtained from a compression isotherm on a Hg/CH_3_CN interface in an electrochemical Langmuir trough. Polarized modulation infrared reflection–absorption spectroscopy (PM-IRRAS) of self-assembled monolayers provided information concerning the average orientation of the rotator with respect to the gold. Air stability measurements of such monolayers on gold showed that the sulfur-containing tentacles start to be detectably oxidized within hours, as determined by XPS. These oxidized molecules can then be washed away in a polar solvent. Detailed molecular dynamic simulations of the altitudinal rotor on a gold surface using the universal force field (UFF) potential, showed synchronous and half-synchronous unidirectional nature of the rotational hysteresis around the horizontal axle in one MMP/PPM pair of conformational enantiomers. Furthermore, the effect of the metal on the motion of adsorbed surface-mounted molecular rotors has been approximated by using the image charges and by implementing a Langevin molecular dynamics with electronic friction. Each of the rotors can exist as three pairs of enantiomers (PPP/MMM, PMP/MPM, and MMP/PPM), where these symbols correspond to the helical P/M symmetry of both tetraarylcyclobutadienes and the rotator in **52**. In Figure 29 are shown two conformations of the dipolar rotor **52** representing maximal (A, where one of the tentacles are eclipsed by the axle) and minimal (B, where the tentacles are staggered with the axle) rotator–tentacle interaction on the surface. While in conformation B the tentacles do not interfere with rotation and the rotation is energetically possible for all pair of enantiomers, the rotational energy barriers for all three stereoisomers in conformation A exceed 30 kcal/mol and hamper rotation of the rotator on the surface [137]. The surface-mounted altitudinal motors nevertheless remain firmly attached in the desired orientation, apparently due to a direct interaction of their Hg atoms with the gold surface, which is in agreement with the recent results of alkylmercury salts on the gold substrates [138–139].

**Figure 30:**
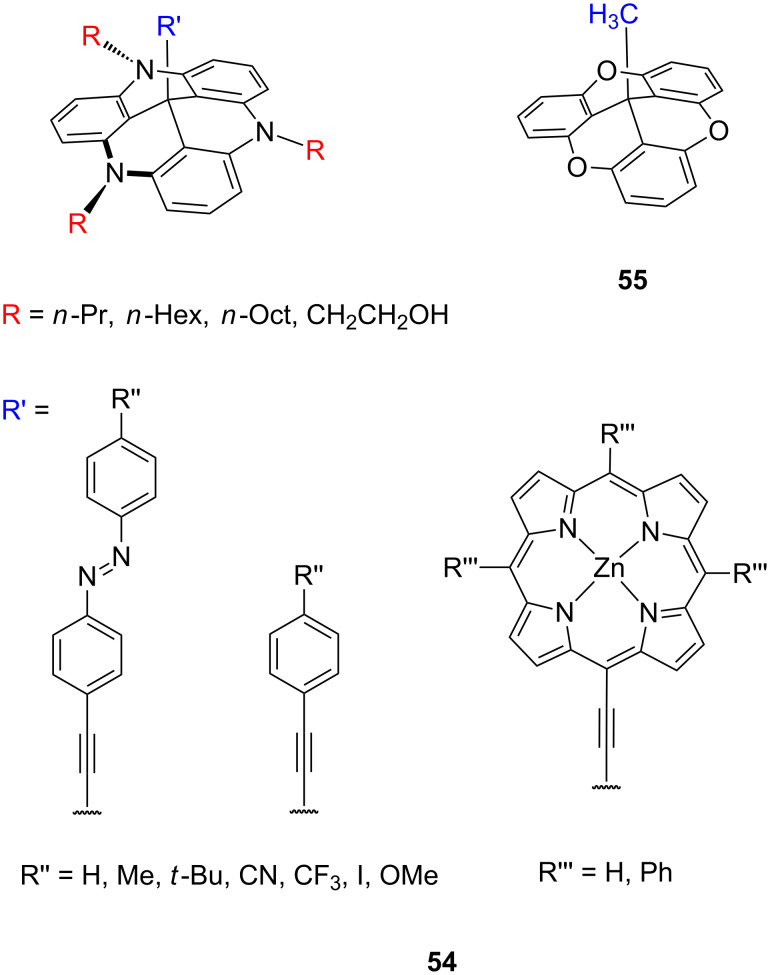
Structure of triazatriangulenium **54** and trioxatriangulenium **55** scaffolds.

Another platforms based on flat triazatriangulenium **54** (TATA) or trioxatriangulenium **55** (TOTA) cation (Figure 30) were synthetized and comprehensively studied by Herges and co-workers [98,140]. The presence of the outer nitrogen atoms and the central carbon atom in the structure of the triangular-shaped TATA platform allowed these molecules to be functionalized either laterally at the edges or vertically at the center and thus serve as a chemically very modular and versatile template to mount functional molecules on the metal surfaces and to form SAMs.

**Figure 31:**
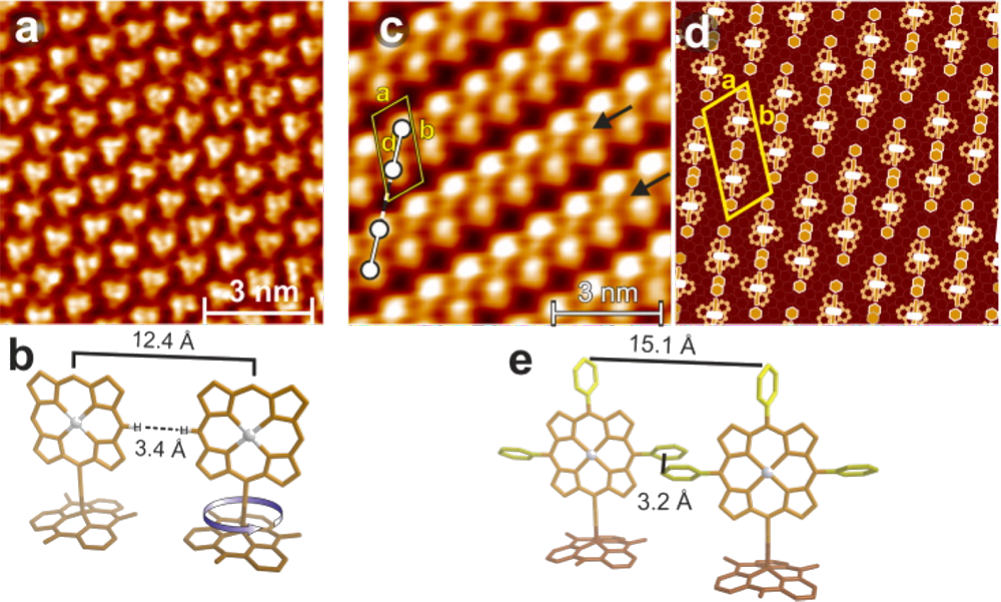
(a) STM image of a porphyrin–TATA adlayer of a zinc-porphyrin derivative of the octyl-TATA platform **54** (R''' = H) on Au(111) (*I*_t_ = 9 pA, *U*_Bias_ = 0.5 V). (b) DFT (PBE/SVP-D2) calculated structure of two neighboring molecules of **54** (R''' = H). The following restraints were applied: distance between neighboring molecules 12.4 Å (taken from STM); only the first four CH_2_ groups of the octyl chains are optimized; all platform nitrogen atoms are in a plane (constraint of the surface); orientation of porphyrins is parallel (barrier of rotation is 0.3 kcal·mol^−1^); all other geometry parameters are optimized. (c) STM image of a triphenylporphyrin-TATA adlayer of **54** (R''' = Ph) on Au(111) (*I*_t_ = 13 pA, *U*_Bias_ = 0.48 V). (d) Structural model of the adlayer. In the unit cell (indicated by the rhombus), there are two molecules separated by a lateral distance *d* = 15.1 Å. (e) DFT (PBE/SVP-D2) calculated structure of two neighboring molecules of **54** (R''' = Ph), at a fixed intermolecular distance of 15.1 Å (for further restraints, see (b)). Reprinted with permission from [145], copyright 2014 American Chemical Society.

This “platform approach” allows for a comprehensive spatial and lateral control of the molecular arrangement and orientation on metal surfaces. The functional moieties attached to the central carbon atom of *C*_3_-symmetrical trioxa- or triazatriangulenium platforms are oriented perpendicular to the metal surface. In the case of TATA platform the size and the lateral arrangement of these platforms in the densely packed hexagonal array on gold is determined by the length of the alkyl side chains at three outer nitrogen atoms. Although, the interaction of functionalized TATA platforms with gold surfaces is mainly based on weak dispersion forces, the binding energy of these platforms is surprisingly higher than that of thiols to gold. It was also found that, despite the presence of a sp^3^-hybridized carbon atom in the conduction path of the TATA unit, the TATA platform exhibits a contact resistance only slightly larger than that of the thiols [141]. The TATA platforms are known to self-assemble into monolayers on Au(111) surfaces, and provide a reliable template for binding a bridging group through the central sp^3^-hybridized carbon atom. Several different derivatives of TATA platforms on Au(111) surfaces uprightly functionalized with ethynyl, phenyl, azobenzene, zinc-porphyrins were synthetized and adlayers have been studied by using STM, XPS, CP-AFM, gap-mode surface-enhanced Raman spectroscopy (SERS), infrared-reflection absorption spectroscopy (IRRAS), cyclic voltammetry (CV), and quantum chemical calculations [142–146]. Using these techniques the TATA platforms were shown to form hexagonally ordered adlayers flatly sitting on the metal substrate where the lattice constant depends on the length of the side chains attached to the nitrogen atoms of the TATA platform and increases from 10.7 Å for propyl to 12.6 Å for octyl side chains (Figure 31). The large footprint of a bulk TATA platform (more than 150 Å^2^) hampers the lateral interactions between the perpendicular functional moieties in the monolayer. In several studies, Herges and Magnussen addressed the features and advantages of these TATA platforms, in particularly to control the spatial arrangement of functional molecules on metal surfaces and to create a free volume for sterically demanding operations in densely packed self-assembled monolayers, e.g., in surface-mounted molecular switches based on azobenzenes [144,146], azimuthal rotors or light-harvesting systems (porphyrins) on metal surfaces [145]. The “platform approach” is thus a suitable method to prepare self-assembled monolayers of functional molecules on, e.g., gold with control of intermolecular distances.

## Conclusion

In this review several approaches to control the spatial arrangement of molecular structures at planar solid substrates have been presented. In several cases the control over the lateral order of these large footprint structures was equally interesting. The main focus was set on tripodal organic architectures and, to the best of our knowledge, organic model compounds exposing three anchor groups for noble metal substrates are discussed comprehensively. In contrast to that, alternative concepts profiting, e.g., from an even larger number of anchor groups or from the van der Waals interaction of extended aromatic systems are only represented with particular appealing examples.

While all platforms exposing multiple anchor groups exhibited an increased stability of the molecular monolayer compared with analogues comprising only a single anchor group, the extent of anchor groups really forming covalent bonds with the substrate and thus also the perfection of structural control at the interface varies considerably between the various design concepts. Also other important structural features such as the spatial control over the protruding molecular subunit, the nature of its coupling to the substrate, or the lateral arrangement of neighboring molecules vary considerably between the different presented examples. The same is also true for chemical aspects such as the modularity of the approach defined by the ease to alter the protruding subunit for a particular footing structure.

The large variety of the different molecular platforms is not surprising because they were not all optimized for the same purpose, and the requirements for an ideal footing structure differ considerably between different applications and are in some cases even in contradiction with each other. While for example a strong electronic coupling between the substrate and the protruding subunit is preferable for electronic devices, it leads to immediate quenching of a molecular excited state in labeling applications and thus, insulating features are more appealing for the latter.

In spite of the large number of already synthesized and investigated multivalent molecular platforms, there remains a rich structural variety to explore. The structure–property correlations of the model compounds reported so far reveal molecular design rules supporting the further development of molecular footing structures. The ideal platform for a particular application still has to be custom-built and we are looking forward to many interesting structures still to be found.
